# Buccopharyngeal route administered high polyphenolic olive oil and COVID‐19: A pilot clinical trial

**DOI:** 10.1002/iid3.1054

**Published:** 2023-10-20

**Authors:** Francisco Rodríguez‐Argente, María Alba‐Domínguez, María P. Díaz‐Martínez, Cristian Díaz‐Vergara, Belén Díaz‐Márques, Paloma Ferrero‐Ortega, Ana C. Gil‐Adrados, Lorena Gómez‐Bernardo, Laura Gordo‐Murillo, Elsa Humanes‐de la Fuente, Jesús Jurado‐Palomo, Ángel Ortega‐González, Juana Machado‐Gallas, Álvaro Moreno‐Ancillo, Gerardo Ávila‐Martín, Ana C. Marín‐Guerrero, Joaquín Álvarez‐Gregori

**Affiliations:** ^1^ Hospital Universitario Nuestra Señora del Prado Talavera de la Reina Spain; ^2^ HistoBioMol Laboratory Lugo Spain; ^3^ Gerencia de Atención Integrada Talavera de la Reina‐Hospital Nuestra Señora del Prado Talavera de la Reina Spain

**Keywords:** immune evasion, immune surveillance, immunomodulation, immunotherapy, mucosal immunity, nasopharyngeal lymphoid associated tissue, olive polyphenols, oromucosal route, SARS‐CoV‐2 infection

## Abstract

**Introduction:**

Waning immunity after vaccination justifies the need for additional effective COVID‐19 treatments. Immunomodulation of local immune response at the oropharyngeal mucosa could hypothetically activate mucosal immunity, which can prevent SARS‐CoV‐2 main immune evasion mechanisms in early stages of the disease and send an effective warning to other components of immune system. Olive polyphenols are biologically active compounds with immunomodulatory activity. There are previous studies based on immunomodulation with olive polyphenols and respiratory infections using an enteral route, which point to potential effects on time to resolution of symptoms. The investigators sought to determine whether participants following immunomodulation with tiny quantities of high polyphenolic olive oil administered through an oromucosal route could have a better outcome in COVID‐19.

**Summary:**

This pilot clinical trial investigated the effect of buccopharyngeal administered high polyphenolic olive oil on COVID‐19 incidence, duration, and severity.

**Importance:**

Waning immunity after vaccination justifies the need of further research for additional effective treatments for COVID‐19.

**Objective:**

Immunomodulation of local immune response at the buccopharyngeal mucosa could hypothetically activate mucosal immunity, which would in turn difficult SARS‐CoV‐2 immune evasion mechanisms in early stages of the disease and send an effective warning to other components of immune system. Olive polyphenols are biologically active compounds with immunomodulatory activity. There are previous studies based on immunomodulation with olive polyphenols and respiratory infections, using an enteral route, which suggest potential shortening of time to resolution of symptoms. The investigators sought to determine whether participants following immunomodulation with tiny quantities of high polyphenolic olive oil administered through an oromucosal route could have a better outcome in COVID‐19.

**Design, Setting, and Participants:**

Double blind, randomized pilot clinical trial conducted at a single site, Talavera de la Reina, Spain. Potential study participants were identified by simple random sampling from the epidemiological database of contact patients recently diagnosed of COVID‐19 during the study period. A total of 88 adult participants were enrolled and 84 completed the 3‐month study, conducted between July 1, 2021 and August 31, 2022.

**Intervention:**

Participants were randomized to receive oromucosal administered high polyphenolic olive oil, 2 mL twice a day for 3 months or no treatment.

**Main Outcome and Measures:**

Primary outcomes were incidence, duration, and severity of COVID‐19 after intervention.

**Results:**

There were no differences in incidence between both groups but there were significant differences in duration, the median time to resolution of symptoms was 3 days in the high polyphenolic olive oil group compared with 7 days in the no‐treatment group. Although time to resolution is directly related to severity, this study did not find any differences in severity.

**Conclusion and Relevance:**

Among full‐vaccinated adults recent infected with COVID‐19, a daily intake of tiny quantities of oromucosal administered high polyphenolic olive oil before infection significantly improved the time to symptom resolution. This finding strongly support the appropriateness of further deep research on the use of oromucosal administered high polyphenolic olive oil as an effective immune strategy against COVID‐19.

## Graphical abstract

This pilot study revolves around the feasibility of a novel immune strategy against COVID‐19 consisting of the buccopharyngeal administration of olive polyphenols (OP). SARS‐CoV‐2 ability to silence the immune surveillance function played by mucosal innate immunity through dendritic cells (DC) could be hypothetically counteract by local immunomodulation with OP.

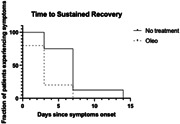

## 1 INTRODUCTION

For infectious diseases where immunization offers lifelong protection the sufficiency of vaccines as a sole effective control method is obvious, however for many diseases caused by respiratory virus, including severe acute respiratory syndrome coronavirus 2 (SARS‐CoV‐2) infection, immunity wanes over time after vaccination.^1^ Vaccines usually produce, through the training of adaptive immunity, an immune response similar to that produced by natural infection.^2^ Despite its indisputable merits, adaptive immunity alone is not sufficient to overcome the challenge of certain infections as coronavirus disease 2019 (COVID‐19).^3^ In fact most immune responses are a combination of different overlapping innate and adaptive mechanisms. It is worth remembering that the first stage of any immune response is the recognition that a foreign agent has entered the body and this key function, more accurately known as immune surveillance, rest in the hands of the innate immunity. Immune surveillance by innate immunity is imperative to early detect any invader so it can be inactivated or eliminated before it causes any harm.^4^

SARS‐CoV‐2 immunopathogeny hits precisely at this sensitive point. Two main obstacles hinder a correct immune surveillance of SARS‐CoV‐2 by the immune system, lung structure itself^5^ and SARS‐CoV‐2 special ability to blunt the master regulator of the innate response to viruses, the interferon (IFN) system.^6^ In relation to lung structure, because T memory (L_TEM_) and long‐lived plasma cells (LLPC) cannot last on the extremely thin pulmonary exchange surface, efficient protection from respiratory viruses after first encounters, must be achieved by a complementary mechanism that consists on the innate immunity giving warning to the adaptive immunity cells, so they can get to the respiratory mucosa in time.^5^ But the efficacy of an immune response does not rely only on the place where the infection takes place but on the nature of the infectious agent too. In this way, SARS‐CoV‐2 dysregulates the IFN system so effectively, that it often disables the immune surveillance function played by the innate immunity mechanisms of the upper respiratory tract mucosa with deleterious consequences for the host.^7^ As a matter of fact this upper respiratory mucosal immunity is a kind of immune watch tower of lung immunity. So in many circumstances (e.g., elder people, immunodeficiency, or as a result of transient immune decay) it does not matter how well‐trained the adaptive immunity against SARS‐CoV‐2 is as the virus silence the warning signal needed to activate an effective immune response.

Children and young adults experience less severe COVID‐19 than aged people because among other causes they have a basal preactivated innate immunity which protect them from severe disease.^8^ Importance of immunosenescence, immunodeficiency, and transient immune decay in apparently healthy individuals are well‐known risk factors in the pathogeny of COVID‐19, mainly because of the impairment of innate immune responses.^9^

The investigators wondered whether it would be possible to counter SARS‐CoV‐2 ability to silence the immune surveillance function played by innate immunity by local oromucosal immunomodulation with high polyphenolic olive oil. Immunomodulation of local immune response at the buccopharyngeal mucosa could hypothetically activate mucosal immunity, which can prevent SARS‐CoV‐2 main immune evasion mechanisms in early stages of the disease. Olive polyphenols are well‐known, pharmacologically active compounds with immunomodulatory activity.^10^ There is previous experience with the use of olive polyphenols in upper respiratory virus infections via enteral (immunomodulation of gut associated lymphoid tissue [GALT]) with potential effect on reduction of time to resolution of symptoms.^11^ Polyphenols are known to modulate immune responses in both the innate and adaptive systems.^12^ There are no previous studies of specific local oromucosal immunomodulation [immunomodulation of NALT]) with olive polyphenols in respiratory tract infections.^13^ The use of the oromucosal route would allow the immunomodulatory substance to work effectively at the portal of entry of SARS‐CoV‐2 which is crucial to efficiently counteract SARS‐CoV‐2 immunopathogeny.^14^ This study attempts to clarify whether buccopharynx mucosal immunomodulation with tiny quantities of high polyphenolic olive oil is a valid immune strategy to mitigate clinical symptoms of COVID‐19.

## 2 MATERIALS AND METHODS

### 2.1 Study design and patients

This study was approved by the independent ethics committee of the Hospital Nuestra Señora del Prado, Spain (Clinical study approval number 22/21) and registered at Trial registration website ClinicalTrials.gov. (Trial registration Identifier: NCT05685901). It was conducted in accordance with the Declaration of Helsinki and Good Clinical Practice guidelines. Written informed consent was obtained from all patients.

This double‐blind, pilot randomized clinical trial of buccopharyngeal mucosa administered high polyphenolic olive oil versus no therapy was conducted from July 1, 2021 to August 31, 2022, coordinated by the Pediatric Department of the Hospital Universitario Nuestra Señora del Prado. Participants were selected from the epidemiological database of nearly 7900 contacts of recent COVID‐19‐diagnosed people with a positive result from a SARS‐CoV‐2 reverse transcriptase–polymerase chain reaction or antigen test performed at the Laboratory of the public Hospital Nuestra Señora del Prado belonging to the National Health system at the city of Talavera de la Reina, Spain.

Adult men and nonpregnant or breast‐feeding women were eligible if they had been recent contacts of people diagnosed of COVID‐19 and had no symptoms nor had a positive result for SARS‐Cov‐2 infection. Patients were excluded if they were symptomatic, if they had had COVID‐19 in the previous 6 months or if they had participated in another clinical trial related to COVID‐19. More details of the trial can be found in the protocol (Supporting Information S1).

### 2.2 Randomization

Participants in the study were randomly allocated in two groups by simple alleatorization using the tool Epidat, program for epidemiological data analysis. Version 4.2, July 2016. Consellería de Sanidade, Xunta de Galicia, Spain; Organización Panamericana de la Salud (OPS‐OMS); Universidad CES, Colombia. Participants were randomly allocated to olive oil group (extent equivalent of 2 mL of early harvest olive oil, containing 0.75 mg of polyphenols) or no treatment group. Allocation assignment was concealed from investigators and patients.

### 2.3 Interventions

Study patients self‐administered 0.75 mg of polyphenols in 2 mL of high polyphenolic olive oil twice a day for 3 months. High polyphenolic olive oil is normal olive oil obtained from olives before they complete full maturation. Its high content in polyphenols gives it a special bitter taste. Early harvest olive oil has been an appreciated product since ancient times and has been part of staple food for millennia in the Mediterranean Basin. Early harvest olive oil used for buccopharyngeal administration was provided by Oleo Toledo group in bottles 500 mL with full standards for human consumption. The olive oil used in the study contained 406 mg of polyphenols per kg of olive oil according to the laboratory determination by the Laboratory of Eurocaja Rural (INE/0451/21). Patients were asked to take the investigational product on an empty stomach and retain it for a couple of seconds in the mouth before swallowing. Participants were advised that they would experience a characteristic scratchy feeling and bitter taste at their throat and mouth produced by the natural high content of polyphenols in early harvest olive oil.

### 2.4 Procedures

A study physician contacted potential study participants by telephone to verify eligibility and obtain informed consent. Patients were then sent enrollment documentation, informed consent, and dispensing of the olive oil. High polyphenolic olive oil was left with participants for self‐administration on Days 1–90. Subsequently, patients were contacted by telephone by study staff on Days 15–30, 60, and 90 for a structured interview. Diagnosis of COVID‐19 in participants who were infected during the study was made by SARS‐CoV‐2 reverse transcriptase‐polymerase chain reaction performed at the laboratory of the Hospital Nuestra Señora del Prado, belonging to the National Health system. Nasophryngeal swabs were tested on the same day of collection from individuals by the Xpert Xpress SARS‐CoV‐2 test, a rapid real‐time RT‐PCR test intended for the qualitative detection of nucleic acid from the SARS‐CoV‐2 in upper respiratory specimens. A study physician reviewed medical records of patients to complete the information required by the protocol.

### 2.5 Outcome measures

The primary outcome was to determine the effect of oromucosal administered high polyphenolic olive oil on COVID‐19 incidence, duration, and severity.

### 2.6 Statistical analysis

#### 2.6.1 Sample size determination

The authors estimated a study sample of 57 patients (28 in each group), this sample size would guarantee 80% power to detect unforeseen problems, such as ambiguous eligibility criteria or misinterpretations of questionnaire items, with a two‐sided type one error rate of 5%. The research team was finally able to recruit 88 patients of which 84 completed the study, which exceeded the minimum sample size calculated after applying the sample calculation formula proposed by Viechtbauer.^15^

#### 2.6.2 Primary outcomes

The primary outcomes were defined as incidence, duration, and severity. Participants' characteristics were collected and presented as means and standard deviations where appropriate. Data for incidence were reported as the odds ratio (OR) with a 95% confidence interval, using a tow‐side Fisher's exact test with significance reported it *p* < .05. The duration was defined as the time the first symptom appeared until complete resolution of symptoms. Patients were analyzed according to the treatment they received. The primary end point of time from of clinical symptoms to complete resolution of symptoms with high polyphenolic olive oil versus no treatment was assessed by a Kaplan–Meier plot and compared with a log‐rank test. Data were analyzed via GraphPadPrism (version 7.03, GraphPad Software, www.graphpad.com).

## 3 RESULTS

### 3.1 Patients

**Figure 1:**
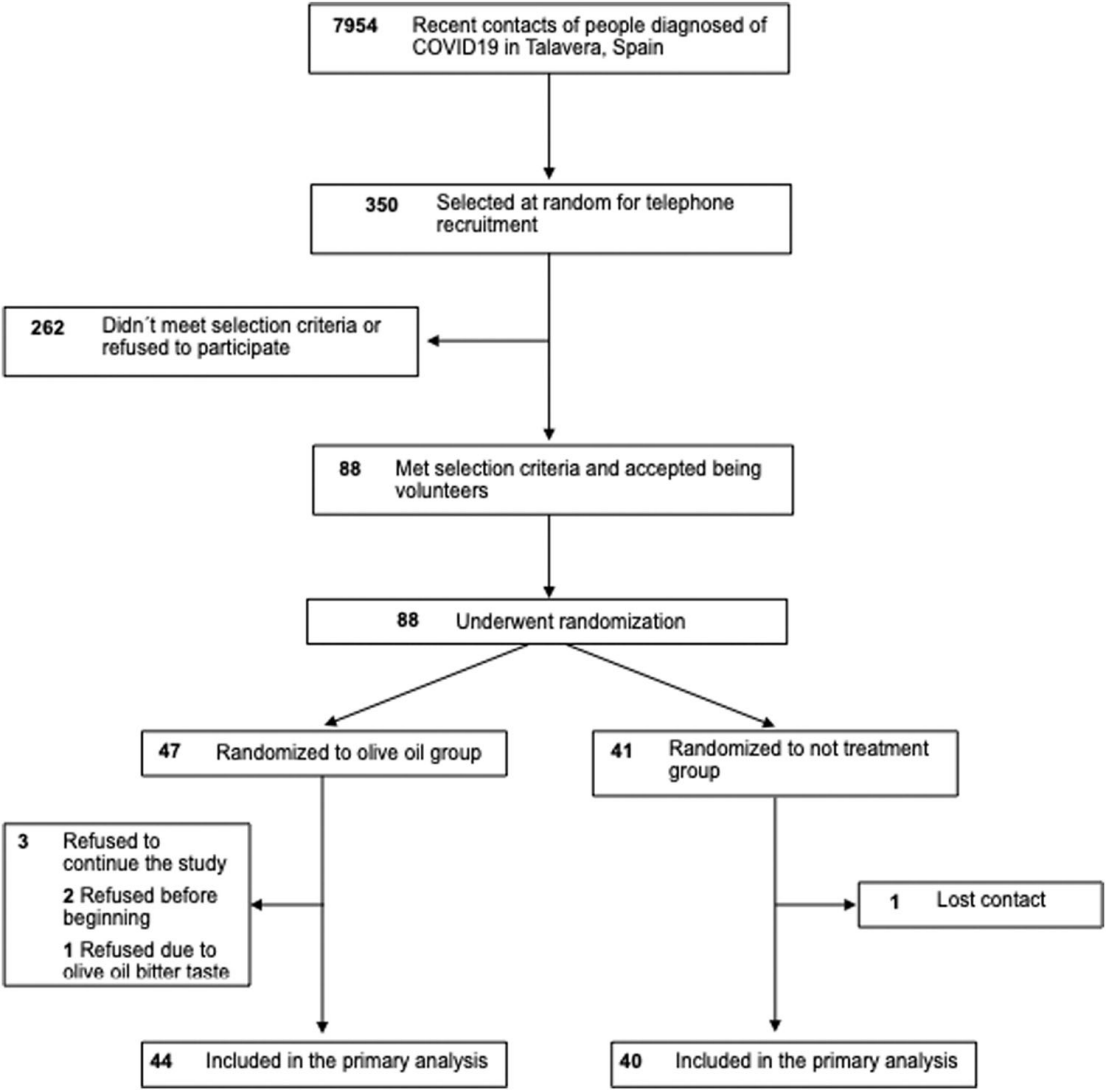
Flow chart.

Of the 88 participants who finally underwent randomization, 84 completed the study between July 1, 2021 and August 31, 2022, 44 were assigned to receive high polyphenolic olive oil and 40 to receive no treatment (Figure 1). Participants in both groups were balanced in demographic characteristics. The median age of patients in the primary analysis population was 51 years (interquartile range [IQR]: 35.5–56.5), 61 (72%) were women, and 23 (28%) were men. No one of them had any known comorbidities at baseline. One hundred percent of participants reported having been vaccinated with at least two doses of a SARS‐CoV‐2 vaccine (Table 1).

**Table 1:** Demographic characteristics of the patients at baseline.

	No. (%)	
Characteristic	Oleo (*n* = 44)	No treatment (*n* = 40)
Age, median (interquartile range), year	52 (35.5–56.5)	51 (40–57.75)
Age groups, years		
<40	15 (34)	9 (23)
40–64	25 (57)	25 (62)
≥65	4 (9)	6 (15)
Sex		
Male	16 (36)	10 (25)
Female	28 (64)	30 (75)
COVID‐19 vaccination	44 (100)	40 (100)

### 3.2 Primary outcomes

**Figure 2:**
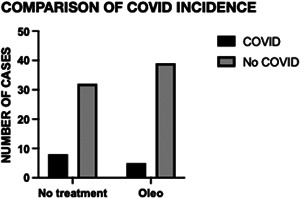
Histogram of COVID incidence in both groups: oleo and no treatment. There were 84 participants in the study. COVID incidence in Oleo group was 6 (proportion 0.11), COVID incidence in control group was 8 (proportion 0.2). There was no significant difference in incidence. Fisher's exact test, *p* = .3683.

**Figure 3:**
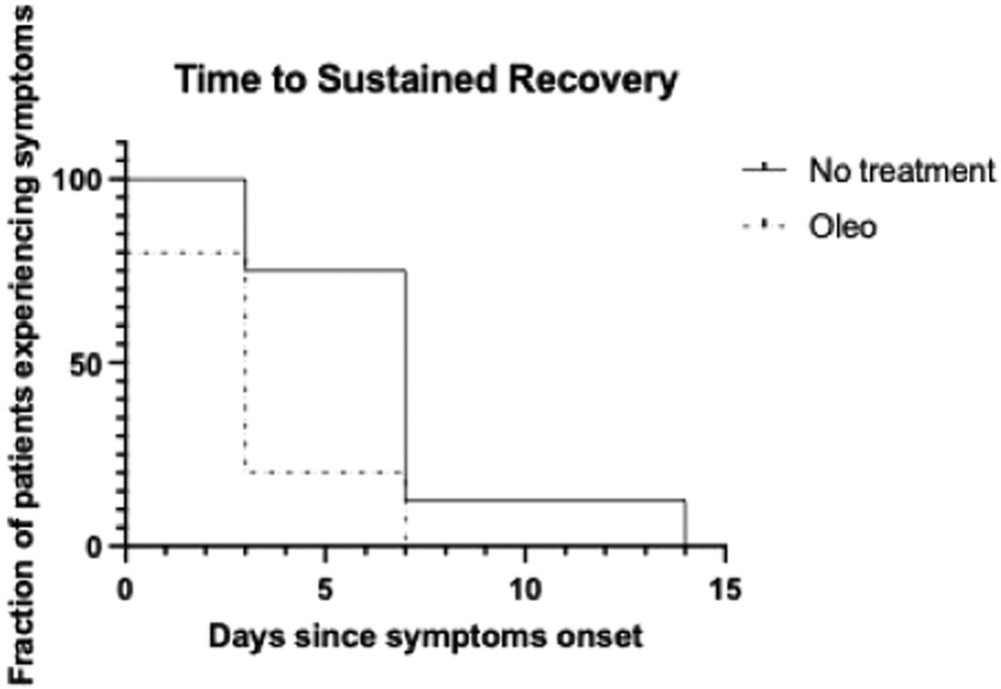
Kaplan–Meier plot. Time to sustained recovery. Recovery was defined as first day without symptoms. All patients were followed up until recovery. Median time to recovery was 3 days in the Oleo group and 7 days in the no‐treatment group. Time to resolution of symptoms in patients assigned to olive oil versus no treatment was significantly different (median, 3 days vs. 7 days; difference, 4 days; HR for resolution of symptoms, 7.11 [95% confidence interval, 1.066–47.46]; *p* = .0428).

In this pilot randomized clinical trial that included 84 patients, there was no significant difference in incidence (Table 2 and Figure 2). However, time to resolution of symptoms in patients assigned to olive oil versus no treatment was significantly different (median, 3 days vs. 7 days; difference, 4 days; HR for resolution of symptoms, 7,11 [95% CI: 1.066–47.46]; *p* = .0428) (Table 3 and Figure 3). This pilot study detected at least a 1.066 HR. In the olive and no treatment groups, symptoms resolved in 80% and 25% of patients, respectively, by Day 3. There was no significant difference in the proportion of patients who got severe symptoms based on the WHO scale. Most common symptoms in both groups were mild illness (Table 4). Only one asymptomatic case was recorded (in the intervention group). There was one possible case of severe illness in the no‐treatment group, defined as oxygen saturation below 94%, but this person was a retired health worker who stayed home (cared by her relatives) and did not need hospitalization for oxygen therapy so she could not be categorized as a severe case based on the WHO criteria.^16^ There were no deaths or hospitalizations in either participant group. In the intervention group, most common symptoms were malaise, low fever, myalgias, and sore throat. The most common symptoms in the control group were malaise, fever, myalgias, and headache. Loss of sense of smell and loss of taste were recorded in 33% of COVID‐19 in the control group, only one case, 20%, of loss of sense of smell and loss of taste was recorded in the intervention group. There is disagreement about the mildness nature of anosmia and ageusia in COVID‐19. Given that both symptoms are associated with viral persistence and inflammation in human neuro‐olfactory cells,^17^ additional studies should be done to clarify this issue.

**Table 2:** Effect of olive pil (oleo) on COVID disease incidence.

	No. (%)		
Characteristic	Oleo (*n* = 44)	No treatment (*n* = 40)	*p*‐Value^a^
Incidence	5 (11)	8 (20)	.3683

^a^ The *p*‐value was calculated with the Fisher's exact test.

**Table 3:** Common symptoms registered in COVID‐19‐positive participants.

	No. (%)	
Symptoms recorded	Oleo (*n* = 5)	No treatment (*n* = 8)
Asymptomatic	1 (20)	0 (0)
Malaise	4 (80)	8 (100)
Low fever	2 (40)	3 (38)
Fever	1 (20)	4 (50)
Odynophagia	2 (40)	4 (50)
Coughing	2 (40)	4 (50)
Headache	0 (0)	3 (38)
Anosmia and ageusia	1 (20)	3 (38)
Myalgias	2 (40)	5 (63)
Oxygen saturation below 94%	0 (0)	0 (0)
Hospitalizations	0 (0)	0 (0)
Deaths	0 (0)	0 (0)

**Table 4:** Outcomes in the primary analysis population.

	No. (%)			
Characteristic	Oleo (*n* = 5)	No treatment (*n* = 8)	Adjusted hazard ratio (HR) (95% credible interval)^a^	*p*‐Value
Resolution of symptoms^a^				
Time to resolution of symptoms, median No. of days (interquartile range)	3 (3–3)	7 (3–7)	7.1 (1.066–47.46)^b^	.0428
Symptoms resolved at 3 days	4 (80)	2 (25)		

^a^ Resolution of symptoms was defined at the first day free of symptoms.

^b^ Hazard ratio for resolution symptoms was estimated by the Mantel–Haesnzel model. The *p*‐value for this ratio was calculated with the log‐rank test.

### 3.3 Adverse events

No adverse events related to the intake of high polyphenolic olive oil were noticed. Only one participant in the treatment group did not continue the study due to the bitter taste of high polyphenolic olive oil.

## 4 DISCUSSION

In this pilot clinical trial among 84 vaccinated people the buccopharyngeal mucosal administration of 2 mL of high polyphenolic olive oil twice daily (equivalent extent of 0.75 mg of olive oil polyphenols twice daily) compared with no treatment significantly shortened time to resolution of symptoms. These findings suggest that the use of oromucosal administered high polyphenolic olive oil has a high potential to be an effective immune strategy to shorten duration of symptoms in mild COVID‐19.

Pilot studies are small‐scale studies that are necessary first steps in exploring novel interventions and novel applications of interventions, this pilot study revolve around the feasibility of a novel immune strategy against COVID‐19 consisting of the buccopharyngeal administration of tiny quantities of high polyphenolic olive oil. In science if one does not first understand the fundamentals of basic research, then there can not be any movement on advanced applications. Which is to say if one does not know to which port one is sailing no wind is favorable. The three main basic principles obtained from fundamental research that have guided this clinical pilot study have been the importance of understanding how respiratory mucosa immunity respond to infection, the immunomodulatory properties of olive polyphenols and the adequacy of the buccopharyngeal mucosal route.

To the authors' knowledge, there are no previous clinical studies on clinical outcomes after buccopharyngeal administration of olive polyphenols in respiratory tract infections.^13^ There are previous studies that suggest some effect of olive polyphenols and other immunomodulatory substances in shortening time to symptom resolution in upper respiratory tract infections but they are based on the use of an enteral route.^11^ On the other hand, there is previous experience of sublingual administration of low doses of interferon alpha, in children with upper respiratory tract infections, that show the feasibility and effectiveness of using an oromucosal route in this clinical scenario.^18^

Mucosal innate immune response key role in early warning the immune system in respiratory tract infection by SARS‐CoV‐2 is a point of fundamental importance.^19^ SARS‐CoV‐2 ability to silence the immune surveillance function played by mucosal innate immunity is neither a minor immunopathological fact. If local mucosa associated tissue immunomodulation could counteract mucosal immunity impairment by viral infections then the buccopharyngeal route would be more effective than the enteric one because of the different homing profile of memory B cells and T cells after induction at these sites.^14^

Many natural and synthetic compounds and medications have immunomodulatory activity, however, only few of them also possess a scientific basis. Olive polyphenols are bioactive substances with immunomodulatory properties via various mechanisms, such as the regulation of mucosal immunity and inflammation.^10^ Polyphenols promote immunity to foreign pathogens via various pathways. They produce an increase in nitric oxide production by macrophages, enhancing their functional activity. Different immune cells express multiple types of polyphenol receptors that recognize and allow cellular uptake of polyphenols, which subsequently activate signaling pathways to initiate immune responses.^20^

While the concept of mucosal innate immunity as an early warning system is not new, we believe its full clinical potential is not been realized. Should mucosal immunomodulation with olive polyphenols be able to overcome viral suppression of mucosa innate host defenses, then it can be a useful strategy in this regard. But it is neither practical nor safe to go directly from basic research to testing on patients. Pilot studies aim to provide a safer transition. This pilot study tries to humbly bridge the gap between basic and clinical research on this issue. Biomedical science is so complex that it is only through collaboration of basic and clinical research that advances can be made. Although a precise definition of the intimate mechanisms that involve innate mucosal immunomodulation by olive polyhenols rest to be done, the clinical data rigorously obtained in this pilot study should not be neglected. To assert that olive polyphenols are able to counteract effectively SARS‐CoV‐2 main immune evasion mechanisms will need for sure of deeper pure research. Such work, while important, exceeds the clinical capacities of the authors and the scope of the study. Nonetheless, this pilot study would have achieved its modest goals if it is able to draw our attention to the appropriateness of further deep research both basic and clinical on the use of buccopharyngeal route administered high polyphenolic olive oil as an effective immune strategy against COVID‐19.

This pilot clinical trial, like other studies conducted in the same time period, has the strength of having been done in the midst of the pandemic with unique epidemiological conditions. Another strength is the time follow‐up, the clinical surveys on participants for 3 months allowed the investigators to get a precise clinical picture of the studied sample. Moreover, the median of time to resolution of symptoms in the control group of our study, 7 days, coincides with the mean duration of symptoms in other published studies with much larger population samples.^21^

There were no significant differences in the proportion of patients who got severe symptoms by the WHO scale in this study. Even so the authors still suspect that the intervention must produce a better outcome given the direct relation between duration and severity of disesases. In any case, a bigger population should be studied to demonstrate this issue.

### 4.1 Limitations

This trial has several limitations. As stated above this is a pilot study, a small‐scale study conducted in preparation for a larger investigation. The purpose of the investigators was to increase the likelihood of a successful future randomized clinical trial by exploring the efficiency, internal validity, and fundamentally, the delivery of proposed trial with a small sample size. Nevertheless, the approach by the investigators has been as rigorous and with the same level of scrutiny as a pivotal trial, including public registration and full ethical concerns. Pilot studies are defined as such owing to the uncertainty about the generalizability of the results they report. Thus, although a larger sample size would be ideal our sample size was sufficient given our power analysis. Second, virological assessments after resolution of symptoms were not performed, but the clinical characteristics that were measured indirectly reflected viral activity and were of interest during the pandemic. Third, asymptomatic cases were underestimated because all the follow‐up was based on clinical data.

## 5 CONCLUSIONS

In this pilot double‐blind randomized trial of completely vaccinated adults with mild COVID‐19, the intake of 2 mL twice daily of high polyphenolic olive oil versus no treatment before infection, succeeded to significantly improving the time to resolution of symptoms. These findings strongly support the appropriateness of further deep research on the use of buccopharyngeal route administered high polyphenolic olive oil in COVID‐19.

## AUTHOR CONTRIBUTIONS

**Francisco Rodríguez‐Argente**: Conceptualization; data curation; formal analysis; funding acquisition; investigation; methodology; project administration; resources; software; supervision; validation; visualization; writing—original draft; writing—review and editing. **María Alba‐Domínguez**: Conceptualization; data curation; formal analysis; investigation; methodology; software; writing—original draft; writing—review and editing. **María P. Díaz‐Martínez**: Data curation; investigation. **Cristian Díaz‐Vergara**: Data curation; investigation. **Belén Díaz‐Márques**: Data curation; investigation. **Paloma Ferrero‐Ortega**: Data curation; investigation. **Ana C. Gil‐Adrados**: Data curation; investigation. **Lorena Gómez‐Bernardo**: Data curation; investigation. **Laura Gordo‐Murillo**: Data curation; investigation. **Elsa Humanes‐de la Fuente**: Data curation; investigation. **Jesús Jurado‐Palomo**: Data curation; investigation. **Ángel Ortega‐González**: Conceptualization; data curation; investigation. **Juana Machado‐Gallas**: Data curation; investigation. **Álvaro Moreno‐Ancillo**: Conceptualization; data curation; investigation; methodology. **Gerardo Ávila‐Martín**: Methodology; project administration; software. **Ana C. Marín‐Guerrero:** Methodology; project administration; software. **Joaquín Álvarez‐Gregori**: Conceptualization; investigation; methodology; project administration; resources. All authors approved the final manuscript as submitted and agreed to be accountable for all aspects of the work.

## CONFLICT OF INTEREST STATEMENT

The authors declare no conflict of interest.

## Supporting information

Supporting information.
