# Supplementary material for: Buccopharyngeal route administered high polyphenolic olive oil and COVID‐19: A pilot clinical trial
Source: Immun Inflamm Dis. 2023 Oct 20;11(10):e1054. doi: 10.1002/iid3.1054 (PMC10587735; doi:10.1002/iid3.1054)
Supplement: Supplementary file 1 — Supporting information. [file IID3-11-e1054-s001.pdf]

ÁREA INTEGRADA
Talavera de la Reina

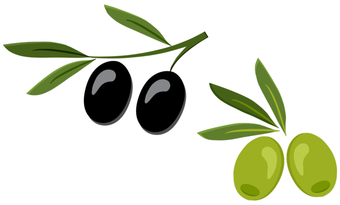

PROTOCOLO DE INVESTIGACIÓN

Ensayo clínico aleatorizado y controlado para la evaluación de la eficacia y seguridad de la inmunoprofilaxis orofaríngea con aceite de oliva rico en polifenoles como atenuación y prevención de la enfermedad infecciosa por el Coronavirus SARS-CoV-2

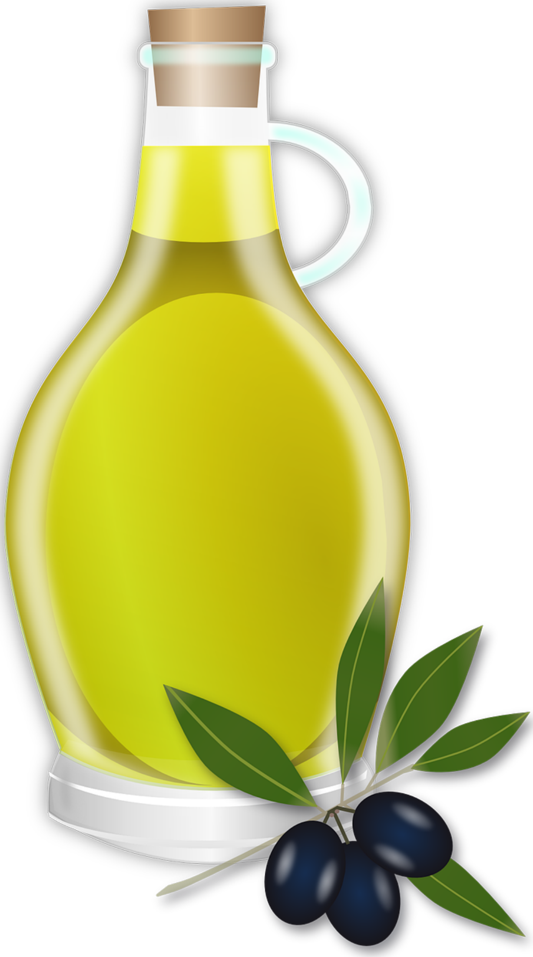

INVESTIGADOR PRINCIPAL

Francisco Rodríguez Argente del Castillo. Gerencia de Atención Integrada de Talavera de la Reina - Hospital Nuestra Señora del Prado (SESCAM). Email: frodrigueza@sescam.jccm.es

INVESTIGADORES COLABORADORES

- **Joaquín Antonio Álvarez Gregori**. Gerencia de Atención Integrada de Talavera de la Reina - Hospital Nuestra Señora del Prado (SESCAM).
- **Álvaro Moreno Ancillo**. Gerencia de Atención Integrada de Talavera de la Reina - Hospital Nuestra Señora del Prado (SESCAM).
- **Ángel Ortega González**. Gerencia de Atención Integrada de Talavera de la Reina - Hospital Nuestra Señora del Prado (SESCAM).
- **Ana Carmen Gil Adrados**. Gerencia de Atención Integrada de Talavera de la Reina - Centro de Salud Río Tajo (SESCAM).
- **Elena Ortiz Muñoz**. Gerencia de Atención Integrada de Talavera de la Reina - Hospital Nuestra Señora del Prado (SESCAM).
- **María Alba Domínguez**. Instituto Kapital Inteligente – Talavera de la Reina.
- **Juana Machado Gayas**. Gerencia de Atención Integrada de Talavera de la Reina - Centro de Salud Río Tajo (SESCAM).
- **María de la Salud Estrada Pastor**. Gerencia de Atención Integrada de Talavera de la Reina - Hospital Nuestra Señora del Prado (SESCAM).
- **Jesús Jurado Palomo.** Gerencia de Atención Integrada de Talavera de la Reina - Hospital Nuestra Señora del Prado (SESCAM).
- **Paloma Ferrero Ortega.** Gerencia de Atención Integrada de Talavera de la Reina – Hospital Nuestra Señora del Prado (SESCAM).
- **María Teresa Núñez** Gerencia de Atención Integrada de Talavera de la Reina - Hospital Nuestra Señora del Prado (SESCAM).

- **Christian Díaz Vergara.** Gerencia de Atención Integrada de Talavera de la Reina - Hospital Nuestra Señora del Prado (SESCAM).
- **Xavier Calderón Cedeño.** Gerencia de Atención Integrada de Talavera de la Reina - Hospital Nuestra Señora del Prado (SESCAM).
- **Santiago Gómez León.** Gerencia de Atención Integrada de Talavera de la Reina - Hospital Nuestra Señora del Prado (SESCAM).
- **Roberto González Ornia.** Gerencia de Atención Integrada de Talavera de la Reina - Hospital Nuestra Señora del Prado (SESCAM).
- **Elsa Humanes de la Fuente.** Gerencia de Atención Integrada de Talavera de la Reina - Hospital Nuestra Señora del Prado (SESCAM).
- **Belén Díaz Marques.** Gerencia de Atención Integrada de Talavera de la Reina - Hospital Nuestra Señora del Prado (SESCAM).
- **Laura Gordo Gordillo.** Gerencia de Atención Integrada de Talavera de la Reina - Hospital Nuestra Señora del Prado (SESCAM).
- **Paz Díaz Martínez.** Gerencia de Atención Integrada de Talavera de la Reina - Hospital Nuestra Señora del Prado (SESCAM).
- **Lorena Gómez Bernardo.** Gerencia de Atención Integrada de Talavera de la Reina - Hospital Nuestra Señora del Prado (SESCAM).

JUSTIFICACIÓN

Los coronavirus son conocidos por ser una amplia familia de virus causantes de un veinte por ciento de todos los casos de infecciones de vías respiratorias también llamadas catarros. El abanico clínico varía desde la práctica ausencia de síntomas, pasando por leve semiología respiratoria hasta enfermedad aguda grave en grupos específicos (pacientes con enfermedades crónicas o en tratamiento inmunosupresor). Esto es lo que creíamos conocer al menos hasta principios del presente siglo XXI cuando la irrupción de dos nuevas cepas de coronavirus SARS-CoV y MERS-CoV mucho más virulentas que las cuatro especies previamente conocidas -229E, OC45, NL63 y HKU1- puso de relieve la gravedad de la amenaza que suponía para la salud global la difusión en la población de un agente viral infeccioso para el que no existiera memoria inmunológica específica en la población ni tratamiento conocido.

Desgraciadamente los peores pronósticos se han hecho realidad con la aparición repentina de un séptimo nuevo coronavirus responsable del síndrome respiratorio agudo grave en Wuhan, China, en diciembre de 2019. Este nuevo coronavirus, llamado SARS-CoV-2 está actualmente causando un considerable número de muertes, a la vez que pone en jaque nuestros hospitales y demás estructuras sanitarias debido a la falta de inmunidad específica en toda la población mundial. Sin una cura conocida, el tratamiento se basa fundamentalmente en el soporte. La carrera por el desarrollo de una vacuna segura y eficaz se encuentra actualmente en el punto de haberse iniciado la tan ansiada vacunación de los primeros grupos de riesgo en la población con las primeras vacunas aprobadas por las distintas agencias nacionales de medicamentos, se espera que los distintos programas de vacunación alcancen a la mayoría de la población entre el próximo verano y el fin de año 2021, entretanto todos esperamos y deseamos los mejores resultados de las vacunas en el corto, medio y largo plazo.

La necesidad de encontrar un tratamiento o prevención mínimamente eficaces hasta poder contar con una vacuna segura ha sido la base de partida para la realización de distintos estudios de múltiples sustancias como profilaxis de la enfermedad causada por el coronavirus SARS-CoV-2 sin haber hasta la fecha resultados satisfactorios con ninguna de las sustancias probadas.

En este sentido el conocimiento de la inmunopatogenia del nuevo coronavirus y la experiencia previa en el tratamiento y prevención de las infecciones respiratorias nos enseñan que puede haber otras estrategias de inmunoterapia, además de la vacunación, que pueden resultar útiles en atenuar la clínica de la infección y por tanto disminuir el número de personas que enferman gravemente, lo que sería de gran utilidad hasta que la eficacia de las vacunas y la cobertura por estas de buena parte de la población estén totalmente completadas. De este modo sabemos que una respuesta inmunitaria innata efectiva por parte de las mucosas de la vía respiratoria alta frente al coronavirus como para otros virus respiratorios es crucial no solo para contener la infección sino para preparar el camino a la respuesta inmune adaptativa. Una estrategia de inmunoterapia basada en la inmunomodulación de la respuesta inmune local en la mucosa orofaríngea podría hipotéticamente ayudar a nuestras mucosas a contener la infección por el coronavirus y de camino facilitar una alerta temprana al sistema inmune adaptativo por parte de estas, de tal forma que la clínica causada por el coronavirus SARS-CoV-2 no fuera tan grave y de esta forma se pudieran salvar vidas y evitar el colapso de nuestros hospitales y unidades de terapia intensiva.

Muchas sustancias naturales y medicamentos tienen supuestamente propiedades inmunomoduladoras, pero solo unos pocos cuentan con una base clínica e inmunopatogénica consistente. Los polifenoles son compuestos presentes en el mundo vegetal con conocida actividad inmunomoduladora. En el caso de la especie Olea europeae (el olivo), sabemos que sus hojas y el fruto verde tienen un alto contenido en polifenoles (fundamentalmente la

Oleuropeína y el Hidroxitirosol). Aunque en el aceite obtenido del fruto del olivo, el aceite de oliva, la cantidad de polifenoles es menor que en las hojas y el fruto verde, se sabe que el aceite obtenido de ciertas variedades, la variedad Cornicabra por ejemplo, puede contener aún un alto contenido en polifenoles sobre todo en aquellos aceites de recogida temprana.

Distintos estudios de los efectos inmunomoduladores de los polifenoles presentes en el olivo en modelos animales establecen que estos inducen un incremento en la producción de óxido nitroso por parte de los macrófagos de las placas de Peyer aumentando su actividad funcional. De esta forma los polifenoles aumentan el procesamiento del antígeno, la actividad presentadora del mismo por parte de los macrófagos, así como la secreción de citoquinas. Funciones todas ellas esenciales en la respuesta inmune local, que los coronavirus, como otros virus respiratorios tratan de inhibir como una de sus estrategias principales a la hora de evadir la respuesta del sistema inmune. Se sabe que la administración experimental de polifenoles del olivo a atletas jóvenes no disminuye la incidencia de infecciones respiratorias, pero si acorta su duración. También se ha comprobado en modelos animales que la administración de polifenoles produce un aumento de volumen del tejido linfoide asociado a mucosas.

Por lo tanto creemos que hay una base sólida tanto clínica como inmunopatológica para sostener que la administración vía mucosa oral, en ayunas, de una pequeña cantidad de aceite de oliva rico en polifenoles puede tener efecto inmunomodulador de la respuesta inmune local de la mucosa orofaríngea y de este modo tener efecto preventivo y atenuante del espectro clínico de la enfermedad causada por Coronavirus.

OBJETIVO Y FINALIDAD DEL ESTUDIO

OBJETIVO PRINCIPAL

Evaluar la efectividad del tratamiento con aceite de oliva de la variedad Cornicabra de recogida temprana usando la vía bucal para inmunomodular la respuesta inmune local de la mucosa orofaríngea como prevención de la enfermedad causada por la infección por SARS-CoV-2.

OBJETIVOS SECUNDARIOS

1. Determinar la gravedad de sintomatología.
2. Evaluar el tiempo en que los pacientes no presentan síntomas desde el inicio del tratamiento.
3. Analizar los efectos adversos.

DISEÑO DEL ENSAYO

TIPO DE DISEÑO

Ensayo clínico aleatorizado con control sin tratamiento.

INTERVENCIÓN

Los sujetos incluidos en el **grupo experimental** recibirán en su domicilio el aceite de oliva variedad Cornicabra (OleoToledo, D.O.P. Montes de Toledo) junto con un documento explicativo sobre la administración correcta del tratamiento (Anexo III). Además, se adjuntará en el envío la Hoja de Información al Paciente y el Consentimiento Informado que se deberá firmar en momento de la entrega del tratamiento.

La vía de administración será la vía bucal/oromucosa con el objetivo de inmunomodular la respuesta inmune local de la mucosa orofaríngea y su tejido linfoide asociado. Se autoadministrarán 2,7 mL de aceite de oliva rico en polifenoles en ayunas, una cucharilla de postre, cada 12 horas, equivalentes a 5mg de polifenoles cada 12 horas.

Los participantes incluidos en el **grupo control** no recibirán tratamiento placebo, recibirán en su domicilio la misma información que recibirá el grupo de tratamiento incluyendo un folleto informativo con unas recomendaciones para la protección frente a COVID19 (Anexo IV), la Hoja de Información al Paciente y el Consentimiento Informado que se deberá firmar en momento de la entrega del folleto informativo.

TÉCNICA DE MUESTREO

Se realizará un muestreo consecutivo de todos los sujetos que acudan como contactos de casos positivos que tengan como resultado una PCR negativa y con aislamiento domiciliario según el protocolo en vigor.

TAMAÑO MUESTRAL

Se reclutarán 500 sujetos repartidos entre el grupo experimental (N=250) y grupo control (N=250), de todos los rangos de edad por encima de 18 años de edad con seguimiento durante 3 meses.

CRITERIOS DE SELECCIÓN

Criterios de inclusión

Los sujetos deben cumplir los siguientes criterios en el momento de dar su consentimiento para ser reclutados en el estudio y mientras se realice el mismo:

1) PCR negativa para SARS-CoV-2.
2) No presentar síntomas de la COVID-19.
3) Ser mayores de 18 años.
4) Informados de su situación epidemiológicas por las Enfermeras de Vigilancia Epidemiológica (EVE).

5) Firma y fecha del consentimiento informado antes de cualquier actividad relacionada con el estudio.

Criterios de exclusión

El sujeto no debe cumplir ninguno de los siguientes criterios:

1) Participación en otro estudio clínico en los 6 meses anteriores.
2) Mujeres embarazadas o en periodo de lactancia.
3) Incapacidad para contestar a las preguntas de los cuestionarios de contacto y de seguimiento.
4) Incapacidad para la autoadministración del tratamiento.
5) Incapacidad de ingesta vía oral.
6) Alergias, intolerancias al tratamiento propuesto o pautas dietéticas incompatibles.
7) Diagnóstico de cualquier otra patología que a criterio de los investigadores pueda incrementar el riesgo del sujeto.

RECOGIDA DE DATOS

Se solicitará a las enfermeras de vigilancia epidemiológica (EVE) encargadas del seguimiento de los contactos el listado diario de contactos en el área de salud, previa autorización de la Gerencia de Área Integrada de Talavera de la Reina (GAITA). Los sujetos seleccionados habrán sido contactados e informados de su situación epidemiológica por las EVE.

Se contactará por teléfono con los sujetos seleccionados y se explicará en que consiste su participación y si cumple los criterios de selección. Si acepta participar:

1º Se rellenará el cuaderno de recogida de datos (CRD) diseñado para tal efecto con las variables basales (día 2 según plan de trabajo) de cada participante.

2º Los participantes serán aleatorizados en el grupo control o en el grupo experimental.

3º Se contactará con el paciente el día 15, 30, 60 y 90 según plan de trabajo y se completará el CRD correspondientes al seguimiento.

Los datos que se recojan en el CRD estarán asociados a un número de identificación (ID). Este cuaderno se ajustará a los requisitos establecidos en cuanto a integridad, exactitud, fiabilidad y consistencia del proceso. Cada investigador se responsabilizará de asegurarse de la correcta cumplimentación, revisión y aprobación de todos los cuadernos de recogida de datos. En todo momento, el investigador será el responsable de la fidelidad y veracidad de todos los datos en él registrados.

El CRD se cumplimentará en formato papel por parte de los investigadores.

Para evitar que los datos personales se puedan asociar directamente a los datos de salud de los participantes, se creará un registro independiente del CRD donde aparecerá los datos identificativos de cada sujeto (Nombre y apellidos, número de historia clínica y número de teléfono). Esté registro llevará asociado un ID para identificar al paciente en el CRD. Este registro tiene como objetivo facilitar el seguimiento de los participantes por parte de los investigadores que desarrollen el trabajo de campo.

PLAN DE TRABAJO

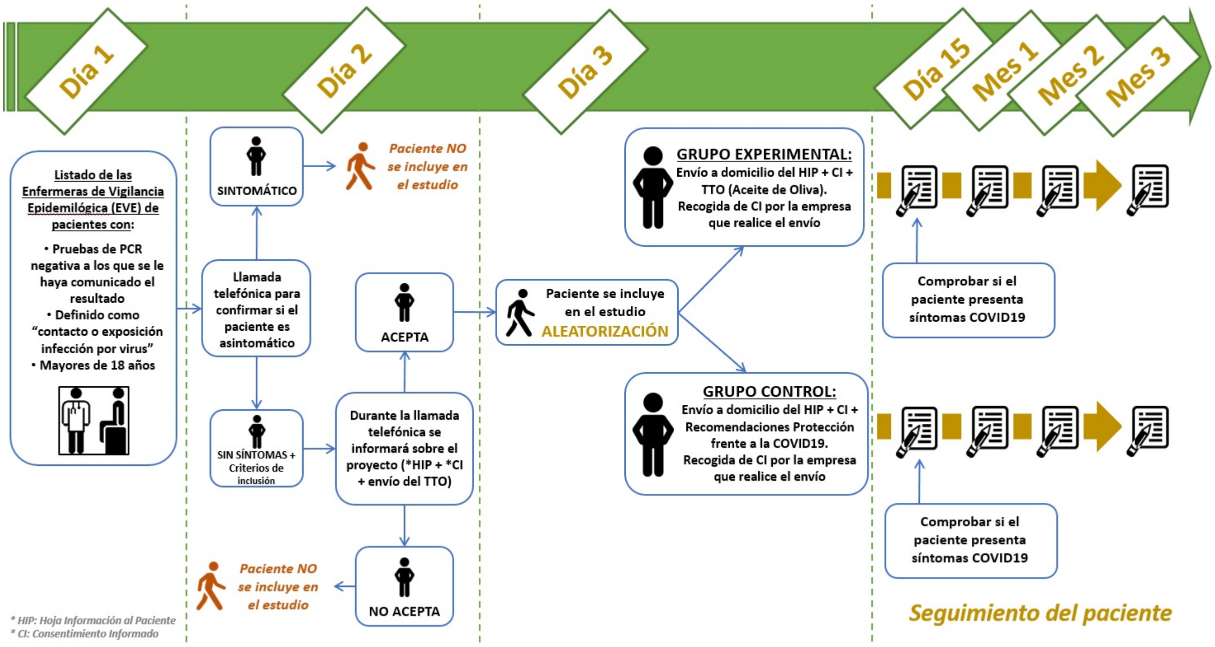

ENMASCARAMIENTO

Dadas las características de la intervención no es del todo posible el enmascaramiento para las pacientes, ni para los investigadores que participan en la intervención o en el grupo control, por lo que se trata de un estudio controlado sin tratamiento.

ALEATORIZACIÓN

Los participantes del estudio se distribuirán de manera aleatoria en dos grupos. Se asignará a los participantes en el grupo experimental o al grupo control, utilizando una aleatorización simple. Para ello se contará con la colaboración de la Unidad de Apoyo a la investigación (GAITA), que realizará la aleatorización utilizando la herramienta Epidat: programa para análisis epidemiológico de datos. Versión 4.2, julio 2016. Consellería de Sanidade, Xunta de Galicia, España; Organización Panamericana de la Salud (OPS-OMS); Universidad CES, Colombia. Con los resultados obtenidos se generarán 500 sobres cerrados que serán abiertos después de la primera llamada (día 2 del plan de trabajo) que contendrán el grupo experimental/control al que corresponde cada participante.

VARIABLES PRINCIPALES Y SECUNDARIAS

Para definir la efectividad del tratamiento descrito en el **objetivo principal** será evaluado como:

Presencia enfermedad durante el periodo de estudio (SI/NO) definida como:

- Paciente hospitalizado con diagnóstico de COVID19.
- Paciente diagnosticado por su médico de Atención Primaria reflejado en su Historia Clínica.

Para evaluar nuestros **objetivos secundarios** se registrarán las siguientes variables:

Gravedad de la enfermedad definida como:

- Leve: asintomático o síntomas respiratorios altos sin neumonía.
- Moderado: neumonía leve sin insuficiencia respiratoria aguda ni respuesta inflamatoria.
- Grave: neumonía con insuficiencia respiratoria aguda, inflamación o hipercoagulabilidad.

Ingreso hospitalario por enfermedad por coronavirus SARS-CoV-2 (SI/NO) y Fecha (ingreso y alta)

Presencia (SI/NO) y duración (nº días) de síntomas clínicos en caso de enfermedad por coronavirus SARS-CoV-2:

- Fiebre/febrícula
- Tos
- Diarrea
- Rinorrea
- Odinofagia
- Mialgias
- Cansancio
- Cefalea
- Anosmia
- Ageusia
- Otros

Eventos adversos: Se tendrá un registro de eventos adversos que pudiesen suceder durante la intervención. Este se mantendrá abierto hasta el final del

estudio. Se informará a los participantes el modo de contactar con el investigador en caso de que sucediese.

Vacunado para COVID19 (SI/NO) y nº de dosis

Variables Sociodemográficas

- Sexo (Hombre/Mujer)
- Fecha de nacimiento
- Peso
- Talla
- Profesión (enseñanza, sanidad, policía, trabajadores cara al público…).
- Grupo de riesgo (ancianos, patología respiratoria de base, paciente psiquiátrico).

Retirada del sujeto (SI/NO) y causa: Los criterios específicos para la retirada de los pacientes serán:

- Toxicidad relacionada con el fármaco, según la valoración del investigador, de severidad o gravedad suficiente para justificar la retirada permanente del tratamiento de estudio.
- Embarazo.
- Incumplimiento de los plazos establecidos.
- Pérdida de seguimiento.
- Fallecimiento por otras causas.

Seguimiento del tratamiento (SI/NO)

RETIRADA DE SUJETOS

Todos los sujetos incluidos en el estudio pueden retirarse en cualquier momento por decisión propia, sin necesidad de justificar su decisión y sin que ello repercuta en sus cuidados médicos. Si un sujeto comunica su intención de abandonar el estudio, o deja de acudir a las visitas programadas, el investigador deberá hacer un esfuerzo razonable para averiguar la razón que le ha llevado a dejar el estudio, siempre y cuando se respeten los derechos del sujeto.

El investigador podrá también retirar a cualquier sujeto incluido por seguridad o si cumple algunos de los criterios de retirada. Si la retirada es debida a un acontecimiento adverso grave, éste deberá ser notificado inmediatamente según los requerimientos de notificación definidos. Los sujetos retirados no podrán volver a ser incluidos en el estudio.

TRATAMIENTO DE LOS SUJETOS

Seguridad del tratamiento del estudio

La forma de administración de los polifenoles bioactivos Oleuropeina e Hidroxitirosol será mediante la administración de pequeñas cantidades de aceite de oliva de la variedad Cornicabra obtenido de la molienda de la aceituna sin ningún aditivo o manipulación adicional, vía mucosa oral. El aceite de oliva es un alimento de consumo habitual en la dieta mediterránea con beneficios nutricionales ampliamente conocidos. Los aceites de oliva para ser considerados ricos en polifenoles deben contar con una concentración de polifenoles mayor de 5 mg por 20 gramos de aceite (250 mg/kg). Y esta cantidad, 20 gramos de aceite es la que reconoce la Unión Europea como la ingesta mínima a partir de la cual se puede esperar efecto antioxidante. Para hacernos una idea 20 gramos vienen a corresponder a una cucharada y media de aceite de oliva, aproximadamente 9 mL. En el estudio actual la ingesta diaria sería de 5mL, la mitad de la ingesta diaria mínima reconocida por la Comisión europea para considerar que un aceite rico en polifenoles pueda tener un efecto antioxidante, único efecto reconocido hasta ahora por las

autoridades europeas responsables de la vigilancia del etiquetado de productos alimenticios como beneficiosos para la salud(Reglamento UE nº 432/2012 de la Comisión).

Medicamento experimental

Aceite de oliva de alto contenido en polifenoles (406 mg por kilogramo de aceite, Cornicabra D.O. de recogida temprana. Informe de Ensayo de Aceite de oliva número INE/0451/21. Laboratorio Eurocaja Rural. Toledo 11 de febrero de 2021). De este modo se administraría 2,7 mL de aceite de alto contenido en polifenoles cada 12 horas (que corresponde a 5 mg de polifenoles cada 12 horas). El aceite de oliva se mantendrá en la boca al menos 20 segundos antes de tragar, dos veces al día, durante 3 meses.

Dispensación

La dispensación del aceite de oliva de alto contenido en polifenoles se realizará mediante envío por correo postal a los domicilios de los participantes en el estudio. El etiquetado del aceite se presentará de la siguiente manera (Figura 1):

Figura 1: *Etiquetado del aceite de oliva utilizado como tratamiento del grupo experimental.*

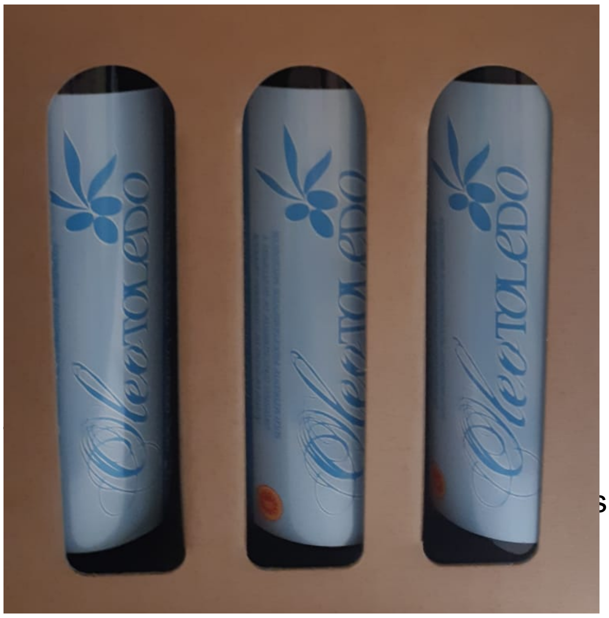

sujetos el control del cumplimiento

ANÁLISIS ESTADÍSTICO

En general, para el grupo control y experimental las variables se resumirán según las siguientes estadísticas descriptivas: número de pacientes, número de pacientes sin datos disponibles, media, desviación estándar, mediana, rango, mínimo y máximo.

La eficacia clínica se evaluará en la visita fin de estudio analizando el porcentaje de sujetos que enfermaron por SARS-CoV-2 durante los 90 días posteriores a la primera administración del tratamiento. Las diferencias de porcentajes entre el grupo control y el grupo de tratamiento se analizarán mediante la prueba de Chi-cuadrado.

Otros análisis secundarios de eficacia serán:

1. Tiempo hasta el desarrollo de la enfermedad: Para describir esta variable se utilizará la media y su rango. Para analizar la diferencia de los tiempos que transcurren entre el inicio de la administración del tratamiento hasta el desarrollo de la enfermedad, se realizará un análisis de supervivencia. Se utilizará el estimador de Kaplan-Mier para realizar este análisis.

2. Gravedad de la enfermedad en ambos grupos: Para valorar si existen diferencias significativas entre los dos grupos en relación a la gravedad de la enfermedad (leve, moderada y severa) se realizará con el test de Chi-cuadrado, o en caso de no ser posible por requisito de aplicación se realizará el Test de la probabilidad exacta de Fisher cuando algún grupo presente valores inferiores a 5. Además, podremos aplicar un test de tendencia lineal.

3. Número de síntomas y duración: Para valorar si existen diferencias entre ambos grupos utilizaremos el test de la T de Student para muestras independientes. En el caso de no cumplirse el requisito de normalidad, se aplicará la prueba U de Mann Whitney.

4. Porcentaje de ingresos: Para valorar si existen diferencias significativas entre los dos grupos en relación al porcentaje de ingresos se realizará con el test de Chi-cuadrado, o en caso de no ser posible por requisito de aplicación se realizará el Test de la probabilidad exacta de Fisher cuando algún grupo presente valores inferiores a 5. Además, podremos aplicar un test de tendencia lineal.

5. Duración del ingreso: Para valorar si existen diferencias entre ambos grupos utilizaremos el test de la T de Student para muestras independientes. En el caso de no cumplirse el requisito de normalidad, se aplicará la prueba U de Mann Whitney.

Para analizar la fuerza de asociación entre la variable dependiente (enfermar o no) e independiente (tratamiento o control “sin tratamiento”), se utilizará un modelo de regresión logística binaria si fuese necesario.

Los análisis ser realizarán según el principio analítico de “intención de tratar”, por el cual se analizarán todos los sujetos aleatorizados de acuerdo con la asignación original de tratamiento o no.

La estimación de los parámetros irá acompañada por el intervalo de confianza al 95% (IC 95%). El nivel de significación estadística se establecerá al 0.05 ($p<0.05$). Para el registro informático de los datos se usará el programa Microsoft Office Excel 2016 ® y para su análisis el programa estadístico SPSS 22.0 (IBM Corp. Released 2013. IBM SPSS Statistics for Windows, Versión 22.0. Armonk, NY: IBM Corp.)

ÉTICA

Información a los sujetos y obtención del consentimiento informado

En el momento de selección, el investigador clínico será responsable de informar de forma completa y en profundidad al sujeto, o a su representante legal de todos los aspectos pertinentes del estudio, naturaleza y objetivos del estudio, así como los posibles riesgos que conlleva, incluyendo la información escrita y el dictamen favorable del comité ético.

El lenguaje utilizado en la información oral vía telefónica no deberá ser técnico, sino práctico y deberá poder ser entendido por el sujeto o su representante legal y el testigo imparcial, cuando proceda. Además, la información no debe contener ningún tipo de lenguaje que lleve a renuncia o a aparecer que renuncia a cualquier derecho legal, o que libere o parezca que libera al investigador, al centro o a su personal de sus obligaciones en caso de negligencia. Ni el investigador, ni el personal del estudio deberán coaccionar o influir indebidamente al sujeto para que participe en el estudio.

Se enviará, junto con el tratamiento del grupo control o experimental, la Hoja de Información al Paciente (Anexo I) y el Consentimiento Informado para su firma (Anexo II).

El estudio se llevará a cabo de acuerdo con los principios éticos de la Declaración de Helsinki, las normas de buena práctica clínica y toda la normativa y legislación aplicable.

Comité ético

Antes del inicio del estudio se habrá obtenido el dictamen favorable, por escrito y fechado, de un comité de ética de la investigación con medicamentos para el protocolo, la hoja de información al paciente, el documento del consentimiento informado, las actualizaciones de este documento, los procedimientos de reclutamiento y cualquier otra información escrita que les sea entregada a los sujetos del estudio.

Durante la realización del estudio se proporcionará al comité ético y autoridades competentes todos los documentos que hayan sido modificados.

Por último, el Comité ético y autoridades competentes serán notificadas del final del estudio y se les presentará un resumen de los resultados y un informe final de acuerdo a las normativas y legislación aplicable.

Confidencialidad de los datos

Todos los datos de los sujetos participantes están protegidos por la legislación de protección de datos aplicable (*Ley Orgánica 3/2018, de 5 de diciembre, de Protección de Datos Personales y garantía de los derechos digitales*). Por ello, todos los sujetos serán identificados por un código de sujeto (ID) que permita su anonimato a todo el personal ajeno al equipo investigador. Este código de sujeto será registrado en un registro de identificación de sujetos donde se relacione dicho código con la historia clínica del sujeto. El registro de identificación de sujetos será custodiado únicamente por el investigador responsable del tratamiento del sujeto, quien deberá proteger la confidencialidad de este registro respetando la privacidad y las normas de confidencialidad de acuerdo con los requisitos legislativos pertinentes.

Los datos originales de los sujetos serán conservados en el centro sanitario y solo tendrán acceso a ellos los investigadores de ese centro para realizar el estudio para el que han sido recogidos. Los investigadores se comprometerán

a no ceder estos datos a terceros. En todo caso, se deberá garantizar la confidencialidad de los datos de los sujetos y velar en todo momento por el cumplimiento de lo recogido en la legislación aplicable.

En los cuadernos de recogida de datos (CRD) no se recogerán nunca datos de carácter personal ni identificativos de los sujetos incluidos. El investigador informará, tal y como recoge la hoja de información al paciente, que los datos obtenidos en el estudio serán guardados y analizados, y que se seguirán las regulaciones aplicables.

LIMITACIONES DEL ENSAYO

SESGO DE RETIRADA DEL PACIENTE

Los pacientes a quienes se les adjudique un tratamiento que no desean, podrían retirarse del estudio al tener conocimiento de ello.

SESGO DEL OBSERVADOR

Dadas las características de la intervención, el estudio presenta una dificultad en el cegamiento de los investigadores. Podría ocurrir que los investigadores encargados del seguimiento pusiesen más énfasis en la recogida de datos de los participantes del grupo de intervención.

SESGO DE SEGUIMIENTO

Podría suceder que la comparabilidad se vea comprometida, si hubiese perdidas en el seguimiento de las pacientes. En el caso de que las perdidas marcaran una diferencia significativa entre los dos grupos, se podría confundir la relación entre la exposición y el resultado.

FINANCIACIÓN Y SEGUROS

Todos los recursos de material fungible necesarios para la realización del proyecto serán financiados por la Fundación Caja Rural.

- Coste aprox. acetite de oliva: 750-1500 euros.
- Coste aprox. gastos de envío de tratamientos: 600-1200 euros.

La recogida de datos se realizará en su gran mayoría durante el horario de jornada laboral de las personas que componen el equipo investigador, como parte sus actividades habituales de seguimiento de pacientes.

INVESTIGADOR PRINCIPAL:

Dr. Francisco Rodríguez Argente del Castillo

- Funciones: Diseño del proyecto. Redacción y corrección del protocolo. Redacción consentimientos informados. Redacción hoja de recomendaciones protección frente al COVID. Solicitud CEIm. Reclutamiento y recogida de datos. Análisis de datos. Difusión de resultados.

MIEMBROS COLABORADORES O ASOCIADOS:

Dr. Joaquín Álvarez Gregori.

- Funciones: Asesoramiento construcción del proyecto. Correción del protocolo. Coordinación y gestión Unidad de apoyo a la investigación y equipo de campo. Gestión colaboraciones. Gestión financiación. Coordinación Fundación Caja Rural. Asesoramiento en el análisis y difusión de los resultados.

Dres. Investigadores colaboradores:

Funciones: Construcción del proyecto. Reclutamiento. Recogida de datos. Análisis y difusión de los resultados.

Unidad de Apoyo a la Investigación

Doctor Gerardo Ávila Martín.

Doctora Ana C. Marín G.

- Funciones: Asesoramiento construcción del proyecto. Corrección y redacción del protocolo. Elaboración y redacción del cuaderno de recogida de datos. Infografía. Aleatorización. Reclutamiento. Análisis de datos y difusión de resultados.

Seguro de responsabilidad civil

Se trata de una intervención dietética de bajo nivel por lo que los daños y perjuicios sobre el sujeto de estudio que pudieran resultar no precisan estar cubiertos por un contrato de seguro o garantía financiera, estando los mismos cubiertos por el seguro de responsabilidad civil profesional colectivo del centro sanitario.

POLÍTICA DE PUBLICACIÓN

El investigador principal del estudio se compromete de forma expresa a publicar toda la información y datos obtenidos tras la finalización del estudio independientemente de su resultado. Al finalizar el estudio, el investigador, cuando proceda, informará al centro y enviará al comité ético un resumen de los resultados del estudio y a la autoridad reguladora los informes que ésta solicite.

Para evaluar de manera cuantitativa la autoría de las publicaciones de los resultados del estudio, se seguirá la normativa publicada por: *A. Acosta. CÓMO DEFINIR AUTORÍA Y ORDEN DE AUTORÍA EN ARTÍCULOS CIENTÍFICOS USANDO CRITERIOS CUANTITATIVOS. Universitas Scientiarum, Vol 12 N° 1, 67-81.*

CRONOGRAMA

2020-2021	Dic	Ene	Feb	Mar	Abr	May	Jun	Jul
Elaboración del proyecto	■	■	■					
Presentación al Comité de Ética de Investigación (CEIm)				■				
Reclutamiento de pacientes					■	■	■	■
Recogida de datos					■	■	■	■
Resultados							■	■
Conclusiones y divulgación de los resultados								■

BIBLIOGRAFÍA

1. Rodríguez-Argente F, Alba-Domínguez M, Ortiz-Muñoz E, Ortega-González Á. Oromucosal immunomodulation as clinical spectrum mitigating factor in SARS-CoV-2 infection. Scand J Immunol. 2021 Jan;93(1):e12972. doi: 10.1111/sji.12972. Epub 2020 Sep 18. PMID: 32892403; PMCID: PMC7816245
2. Greenberg SB. Update on rhinovirus and coronavirus infections. Semin Respir Crit Care Med. 2011;32(4):433-446. - PubMed
3. American Academy of Pediatrics. Coronaviruses, including SARS and MERS In: Kimberlin DW, Brady MT, Jackson MA, Long SS, eds. Red Book: 2018 Report of the Committee on Infectious Diseases. 31st ed. Itasca, IL: American Academy of Pediatrics; 2018:297-301.
4. De la Flor i Bui J. Infecciones de las vías respiratorias altas-1: el resfríado común. Pediatr Integral. 2017;XXI(6):377-398.

5. de Wit E, van Doremalen N, Falzarano D, Munster VJ. SARS and MERS: recent insights into emerging coronaviruses. Nat Rev Microbiol. 2016;14(8):523-534. - PMC - PubMed
6. Zhu N, Zhang D, Wang W, et al. A novel coronavirus from patients with pneumonia in China, 2019. N Engl J Med. 2020;382(8):727-733. - PMC - PubMed
7. Castagnoli R, Votto M, Licari A, et al. Severe Acute Respiratory Syndrome Coronavirus 2 (SARS-CoV-2) infection in children and adolescents: a systematic review [published online ahead of print, 2020 Apr 22]. JAMA Pediatr. 2020;174(9):882. - PubMed
8. Instituto de Salud Global de Barcelona. Instituto de Salud Global Barcelona. COVID-19: Lecciones y recomendaciones, 2018. Disponible en: https://www.isglobal.org/coronavirus-lecciones-y-recomendaciones. 2020/05/24
9. Callow KA, Parry HF, Sergeant M, Tyrrell DA. The time course of the immune response to experimental coronavirus infection of man. Epidemiol Infect. 1990;105(2):435-446. - PMC - PubMed
10. Shi Y, Wang Y, Shao C, et al. COVID-19 infection: the perspectives on immune responses. Cell Death Differ. 2020;27(5):1451-1454. - PMC - PubMed
11. Sungnak W, Huang N, Bécavin C, et al. SARS-CoV-2 entry factors are highly expressed in nasal epithelial cells together with innate immune genes. Nat Med. 2020;26:681-687. - PubMed
12. Murphy K, Weaver C. The mucosal immune system In: Murphy K, Weaver C, eds. Janeway`s Immunobiology: The Immune System in Health and Disease. New York, NY: Garland Science, Taylor & Francis Group, LLC; 2017:493-531.
13. Crespo HJ, Lau JT, Videira PA. Dendritic cells: a spot on sialic Acid. Front Immunol. 2013;4:491. - PMC - PubMed
14. Mubarak A, Alturaiki W, Hemida MG. Middle East Respiratory Syndrome Coronavirus (MERS-CoV): infection, immunological response, and vaccine development. J Immunol Res. 2019;2019:6491738. - PMC - PubMed
15. Channappanavar R, Zhao J, Perlman S. T cell-mediated immune response to respiratory coronaviruses. Immunol Res. 2014;59(1–3):118-128. - PMC - PubMed
16. Xu Z, Shi L, Wang Y, et al. Pathological findings of COVID-19 associated with acute respiratory distress syndrome [published correction appears in Lancet

Respir Med. 2020 Feb 25]. Lancet Respir Med. 2020;8(4):420-422. - PMC - PubMed

17. Ginaldi L, Loreto MF, Corsi MP, Modesti M, De Martinis M. Immunosenescence and infectious diseases. Microbes Infect. 2001;3(10):851-857. - PubMed
18. Mackinnon LT. Exercise and resistance to infectious illness In: Mackinnon LT, ed. Advances in Exercise and Immunology. Champaign, IL: Human Kinetics; 1999:1-26.
19. Palm NW, Medzhitov R. Not so fast: adaptive suppression of innate immunity. Nat Med. 2007;13(10):1142-1144. - PMC - PubMed
20. Wölfel R, Corman VM, Guggemos W, et al. Virological assessment of hospitalized patients with COVID-2019 [published online ahead of print, 2020 Apr 1]. Nature. 2020;581(7809):465-469. - PubMed
21. Livy. Ab urbe condita. Cambridge, MA: Harvard University Press; 1922.
22. Committee on New Directions in the Study of Antimicrobial Therapeutics: Immunomodulation. Promising Approaches to the Development of Immunomodulation for the Treatment of Infectious Diseases: Report of a Workshop. Washington, DC: National Academies Press, 2006. Available from: https://www.ncbi.nlm.nih.gov/books/NBK19846
23. Jesenak M, Urbancikova I, Banovcin P. Respiratory tract infections and the role of biologically active polysaccharides in their management and prevention. Nutrients. 2017;9(7):779. - PMC - PubMed
24. Somerville V, Moore R, Braakhuis A. The effect of olive leaf extract on upper respiratory illness in high school athletes: a randomised control trial. Nutrients. 2019;11(2):358. - PMC - PubMed
25. Brandtzaeg P. Secretory immunity with special reference to the oral cavity. J Oral Microbiol. 2013;5(1):20401. - PMC - PubMed
26. French MA, Moodley Y. The role of SARS-CoV-2 antibodies in COVID-19: healing in most, harm at times. Respirology. 2020;25:680-682. - PMC - PubMed
27. Beilharz MW, Cummins MJ, Bennett AL, Cummins JM. Oromucosal administration of interferon to humans. Pharmaceuticals. 2010;3(2):323-344. - PMC - PubMed

ANEXOS

ANEXO I: HOJA DE INFORMACIÓN AL PACIENTE O REPRESENTANTE LEGAL

Título del Estudio:

"Ensayo clínico aleatorizado y controlado para la evaluación de la eficacia y seguridad de la inmunoprofilaxis orofaríngea con aceite de oliva rico en polifenoles como atenuación y prevención de la enfermedad infecciosa por el Coronavirus SARS-CoV-2."

Promotores del Estudio:

Francisco Rodríguez Argente del Castillo y Joaquín Gregori de los Servicios de Pediatría, Urgencias y la Unidad de Investigación del Hospital Nuestra Señora del Prado de Talavera:

Le invitamos a participar en una investigación sobre uso dietético del aceite de oliva, protagonista principal de la dieta mediterránea, como posible atenuante y preventivo de la enfermedad producida por el Coronavirus. El aceite de oliva rico en polifenoles es un aceite de oliva virgen extra normal obtenido de la aceituna en la almazara sin añadirle ningún aditivo ni químico ni natural; su riqueza en polifenoles se obtiene por ser un aceite obtenido de aceitunas recogidas de primera cosecha o cosecha temprana de una especie singularmente rica en polifenoles, en este caso la especie predominante en nuestra región, la aceituna cornicabra. Los polifenoles por tanto no son añadidos al aceite, sino que el mismo aceite los contiene de forma natural. Este aceite de recogida temprana o rico en polifenoles ha sido y es ampliamente utilizado en nuestra dieta tanto de forma doméstica como en la gastronomía por sus especiales cualidades podríamos decir que durante miles de años. La característica principal del aceite de recogida temprana aparte de su sabor fresco es el amargor y el picor en garganta que producen curiosamente los mismos polifenoles que pretendemos estudiar. El estudio ha sido aprobado por el Comité de Ética de Investigación del Hospital Nuestra Señora del Prado de Talavera. Antes de decidir si desea participar en este estudio, es importante que entienda por qué es necesaria esta investigación, lo que va a implicar su participación, cómo se va a utilizar su información y sus posibles beneficios, riesgos y molestias. Por favor, tómese el tiempo necesario para leer atentamente la información proporcionada a continuación.

¿Cuál es el motivo del estudio?

En este estudio se pretende conocer si la ingesta en ayunas de 2,7 mL (una cucharilla de las de postre) de aceite de oliva rico en polifenoles dos veces al día puede tener efectos atenuantes y preventivos frente a la gravedad de la enfermedad causada por el Coronavirus mediante la inmunomodulación de la respuesta defensiva local de la mucosa oral y faríngea.

Resumen del estudio:

Se propone desarrollar un estudio clínico aleatorizado y controlado dirigido a la prevención y atenuación de la enfermedad por Coronavirus mediante la ingesta de 2,7 mL de aceite de oliva rico en polifenoles dos veces al día en comparación con un grupo no tratado. Se incluirá en el estudio un total de 500 participantes reclutados en el Área integrada de Talavera de la Reina.

Participación voluntaria y retirada del estudio:

Debe saber que su participación en este estudio es voluntaria y que puede decidir no participar o cambiar su decisión y retirar el consentimiento en cualquier momento, sin que por ello se altere la relación con su médico ni se produzca perjuicio alguno en su tratamiento y seguimiento. En caso de que Vd. decidiera abandonar el estudio puede hacerlo permitiendo el uso de los datos obtenidos hasta el momento o, si fuera su voluntad, sus datos borrados de los ficheros informáticos. También se le podrá retirar del estudio si en cualquier momento se le detectase algún tipo de intolerancia o malestar relacionados con la dieta. Todo esto se realizará en todo momento de manera coordinada y bajo la supervisión de un médico.

¿Quién puede participar?

El estudio se realizará en voluntarios mayores de edad de ambos sexos que hayan sido contactos de algún caso positivo a SARS-CoV-2. El reclutamiento de los participantes será a través del sistema de rastreo. Si acepta participar usted va a formar parte de un estudio en el que se incluirán a unos 500 pacientes procedentes de Área integrada de Talavera de la Reina. Tras comprobar que se cumplen los criterios de inclusión para poder empezar el estudio, los participantes del brazo de tratamiento recibirán en su domicilio el aceite de oliva y el consentimiento informado, los participantes del brazo control recibirán el consentimiento informado.

¿En qué consiste este estudio?

El estudio consiste en comparar un tipo de intervención sobre dieta con no intervención. En concreto se pretende ver si la ingesta en ayunas de aceite de oliva rico en polifenoles puede conferir alguna protección o atenuación frente a la enfermedad producida por el Coronavirus. En la primera visita se recogerán datos sociodemográficos, personales y clínicos del participante, edad, sexo, escolarización, antecedentes personales, antecedentes patológicos y toma de medicaciones. En el caso de que usted no cumpliera con los criterios de inclusión propuestos no podría ser incluido en el presente estudio. El estudio compara la intervención que consiste en la ingesta en ayunas de aceite de oliva rico en polifenoles 2,7 mL cada 12 horas durante 3 meses. Aquellos participantes que sean incluidos dentro del grupo de intervención recibirán, de manera gratuita, aceite de oliva virgen extra de la variedad cornicabra. La entrega gratuita de estos productos se hará a domicilio mediante paquetería. La duración del estudio es de 3 meses. Se realizarán visitas telefónicas de revisión a los 0-15-30-60 y 90 días. En cada visita se preguntará sobre evolución, sintomatología… Es importante resaltar que el aceite que recibirá no presenta ningún aditivo ni modificación y que en todo momento usted podrá seguir todas y cada una de las medidas profilácticas y terapéuticas vigentes frente a la enfermedad producida por el Coronavirus sin que el presente estudie contraindique o excluya ninguna de ellas.

¿Cómo se asigna la intervención?

El tipo de intervención que va a recibir se asigna al azar, es decir, ni usted ni el investigador deciden el grupo al que van a pertenecer. La asignación al azar es aceptable porque ambos grupos son igualmente recomendables y no existe ningún ensayo previo sobre el efecto preventivo y atenuante frente a la enfermedad por Coronavirus del aceite de oliva rico en polifenoles. Este

procedimiento es muy necesario para que los resultados del estudio sean válidos.

¿En qué consiste mi participación?

Una vez que ha sido seleccionado su participación consistirá en:

1. Contestar encuestas sobre su estado de salud.

2. Facilitar que se le realice una historia clínica.

3. Autorizar a los investigadores el acceso a su historial médico con el fin de confirmar y actualizar la información médica necesaria para el estudio. Como se le ha explicado previamente usted será seleccionado al azar para participar en uno de los dos posibles grupos de intervención. La participación descrita más arriba no es igual en los dos grupos. La diferencia es que en uno de ellos se le recomendará seguir las prevenciones habituales y la toma de aceite de oliva rico en polifenoles en ayunas y se le proporcionará aceite de oliva virgen extra, y al otro grupo se le indicará seguir las prevenciones habituales solamente.

¿Cómo se accederá a mi historial médico y con qué fines?

Durante el transcurso del estudio los miembros del equipo investigador necesitan poder acceder a su historia clínica para consultar su evolución. Su historia clínica se consultará por un médico según la forma de acceso habitual. En ningún caso se sacará el original de su correspondiente archivo. En caso de ser necesario documentar información obtenida a partir de su historia clínica, se realizará una copia anónima. El equipo de investigadores accederá a su historia clínica por cuenta del responsable del fichero.

¿Cuáles son los posibles beneficios y riesgos derivados de mi participación en el estudio?

Su participación en el estudio le puede ayudar a un mejor conocimiento de su estado de salud, a un mejor control de los factores de riesgo, así como al seguimiento de una dieta saludable adaptada a su estado de salud, así como a su edad y sexo. En trabajos publicados acerca del consumo de los polifenoles del olivo como prevención de otras infecciones respiratorias distintas al Coronavirus se ha demostrado que los polifenoles son capaces de acortar el número de días que duran dichas infecciones respiratorias. Además, es por toda la población conocido que el aceite de oliva de la dieta Mediterránea ayuda a prevenir enfermedades como el infarto o el ictus en personas de alto riesgo cardiovascular y otras enfermedades crónicas. También es posible que usted no obtenga ningún beneficio directo por participar en el estudio. No obstante, se prevé que la información que se obtenga pueda beneficiar en un futuro a otros pacientes y pueda contribuir a un mejor conocimiento del efecto de los polifenoles del aceite de oliva sobre la enfermedad por Coronavirus y otras enfermedades infecciosas respiratorias. Al finalizar la investigación podrá ser informado, si lo desea, sobre los principales resultados y las conclusiones generales del estudio. El estudio no supone ningún riesgo para su salud ya que la cantidad de aceite de oliva ingerida al día será muy pequeña, 5mL al día. Al no tratarse de un estudio con fármacos, no se prevé ningún efecto adverso, a no ser alguna reacción de hipersensibilidad al aceite de oliva, que seguramente usted ya habrá probado alguna vez.

¿Quién tiene acceso a mis datos personales y cómo se protegen?

El tratamiento, la comunicación y la cesión de los datos de carácter personal de todos los sujetos participantes se ajustará a lo dispuesto en la Ley Orgánica 3/2018, de 5 de diciembre, de Protección de Datos Personales y garantía de los derechos digitales. De acuerdo a lo que establece la legislación mencionada, usted puede ejercer los derechos de acceso, modificación, oposición y cancelación de datos, para lo cual deberá dirigirse a su médico del

estudio. Los datos recogidos para el estudio estarán identificados mediante un código y solo su médico del estudio/colaboradores podrá relacionar dichos datos con usted y con su historia clínica. Por lo tanto, su identidad no será revelada a persona alguna salvo excepciones, en caso de urgencia médica o requerimiento legal. Los investigadores le garantizamos que su identidad no trascenderá al equipo clínico. Todos los datos que se obtengan de su participación en el estudio serán almacenados con un código y en un lugar seguro, de acceso restringido. En todo el proceso se seguirá la Ley de Protección de Datos (Ley orgánica 3/2018, de 5 de diciembre) y otras leyes vigentes aplicables. Los investigadores a menudo establecemos colaboraciones con otros investigadores de nuestro país u otros países. En estas colaboraciones los datos que se transmitan en ningún caso contendrán información que le pueda identificar directamente, como nombre y apellidos, iniciales, dirección, nº de la seguridad social, etc. En el caso de que se produzca esta cesión, será para los mismos fines del estudio descrito y garantizando la confidencialidad como mínimo con el nivel de protección de la legislación vigente en nuestro país. El acceso a su información personal quedará restringido al médico del estudio/colaboradores, autoridades sanitarias (Agencia Española del Medicamento y Productos Sanitarios), al Comité Ético de Investigación Clínica y personal autorizado por el promotor, cuando lo precisen para comprobar los datos y procedimientos del estudio, pero siempre manteniendo la confidencialidad de los mismos de acuerdo a la legislación vigente. Si usted lo autoriza, los datos clínicos encontrados durante el estudio y que sean relevantes para su salud le serán comunicados. Estos datos clínicos pueden ser resultados previstos en los objetivos del estudio o pueden ser hallazgos inesperados pero relevantes para su salud.

¿Recibiré algún tipo de compensación económica?

No se prevé ningún tipo de compensación económica durante el estudio. Los participantes en el grupo de intervención recibirán el aceite rico en polifenoles de manera gratuita.

¿Quién financia esta investigación?

Esta investigación se está financiando dentro de la actividad habitual clínico/investigadora de los integrantes del equipo investigador dentro de sus respectivos departamentos, todos pertenecientes al Área integrada de Talavera, recursos propios de los investigadores y la colaboración de la Caja Rural. Está previsto además presentar el proyecto a otras convocatorias regionales, nacionales e internacionales.

Otra información relevante:

Cualquier nueva información referente al estudio y que pueda afectar a su disposición para participar en el estudio, que se descubra durante su participación, le será comunicada lo antes posible. Si usted decide retirar el consentimiento para participar en este estudio, ningún dato nuevo será añadido a la base de datos y, puede exigir la destrucción de sus datos. También debe saber que puede ser excluido del estudio si los investigadores del estudio lo consideran oportuno, ya sea por motivos de seguridad, por cualquier acontecimiento adverso que se produzca o porque consideren que no está cumpliendo con los procedimientos establecidos. En cualquiera de los casos, usted recibirá una explicación adecuada del motivo que ha ocasionado su retirada del estudio Al firmar la hoja de consentimiento adjunta, se compromete a cumplir con los procedimientos del estudio que se le han expuesto.

Destino de los datos:

Los datos recogidos para el estudio se almacenarán en un fichero que se enviará a la Agencia Española de Protección de Datos. Con estos datos los

investigadores hacemos los análisis estadísticos pertinentes para poder extraer los resultados.

Cesión de datos:

Los investigadores a menudo establecemos colaboraciones con otros investigadores de nuestro país u otros países. En estas colaboraciones podemos ceder datos. Siempre lo hacemos siguiendo la normativa legal vigente y para proteger su confidencialidad, estas cesiones siempre se hacen con los datos codificados. Es decir, que ni su nombre ni ningún otro dato identificativo aparezca.

Calidad científica y requerimientos éticos del estudio:

Este estudio ha sido ha sido sometido a aprobación por el Comité Ético de Investigación con Medicamentos. Área integrada de Talavera, que vela por la calidad científica de los proyectos de investigación que se llevan a cabo en el centro. Este comité vigila para que la investigación que se hace con personas se haga de acuerdo con la declaración de Helsinki y aplicando la normativa legal vigente sobre investigación biomédica (ley 14/2007, de 3 de junio de investigación biomédica) y ensayos clínicos BOE-A-2015-14082.

Preguntas:

Llegado este momento le damos la oportunidad de que, si no lo ha hecho antes, haga las preguntas que considere oportunas. El equipo investigador le responderá lo mejor que le sea posible.

Investigadores del estudio:

Si tiene alguna duda sobre algún aspecto del estudio o le gustaría comentar algún aspecto de esta información, por favor no deje de preguntar a Francisco Rodríguez Argente del Castillo. Unidad de Investigación. Hospital Nuestra

Señora del Prado. En caso de que una vez leída esta información y aclaradas las dudas decida participar en el estudio, deberá firmar su consentimiento informado.

ANEXO II: CONSENTIMIENTO INFORMADO

Nombre y Apellidos Participante:

Quién ha informado:

Acompañante (tutor o representante legal):

- ❒ Acepto participar de forma voluntaria en el estudio: "Ensayo clínico aleatorizado y controlado para la evaluación de la eficacia y seguridad de la inmunoprofilaxis orofaríngea con aceite de oliva rico en polifenoles como atenuación y prevención de la enfermedad infecciosa por el Coronavirus SARS-CoV-2."
- ❒ He leído la Hoja de Información al Paciente, comprendo los riesgos y los beneficios que comporta, que mi participación es voluntaria y que me puedo retirar o solicitar que retiren mis datos y/o muestras siempre que quiera.

- ❒ Comprendo que mi participación en el estudio consiste en: Asistir a las visitas programadas y sesiones individuales y/o grupales informativas, seguir con las pautas indicadas por los dietistas/médicos del estudio y consumir los alimentos proporcionados por los investigadores.
- ❒ Doy mi permiso para que los investigadores contacten conmigo nuevamente si soy apto para el estudio a través de los teléfonos que también indico:
- ❒ Doy permiso para ser informado sobre los resultados de las pruebas que me realicen durante el estudio y que sean relevantes para mi salud.
- ❒ Comprendo que no recibiré un beneficio directo por mi participación en este estudio y que no recibiré ningún beneficio económico en el futuro en el caso en que se desarrolle un nuevo tratamiento o test médico.
- ❒ Comprendo que la información del estudio será confidencial y que ninguna persona no autorizada tendrá acceso a los datos o a las muestras.
- ❒ Sé cómo ponerme en contacto con los investigadores si lo necesito.

Firmas Participante:

Acompañante (tutor o representante legal):

Fecha (Día/mes/año):

DENEGACIÓN O REVOCACIÓN DEL CONSENTIMIENTO Después de ser informado/a de la naturaleza del procedimiento propuesto, manifiesto de forma libre y consciente mi DENEGACIÓN/REVOCACIÓN para su realización.

Fdo: El paciente

Fdo: Tutor/ Representante

Fdo: El Profesional

Contacto: En el caso que necesite ponerse en contacto con los investigadores del estudio puede hacerlo a través del Investigador Principal: Dr. Francisco Rodríguez Argente del Castillo. Servicio de Pediatría y Unidad de Investigación. Hospital Nuestra Señora del Prado. Este documento junto con el proyecto ha sido aprobado por el Comité Ético de Investigación con Medicamentos. Área integrada de Talavera.

ANEXO III: DOCUMENTO EXPLICATIVO SOBRE LA ADMINISTRACIÓN DEL TRATAMIENTO

El aceite de oliva rico en polifenoles es un aceite de oliva virgen extra normal obtenido de la aceituna en la almazara sin añadirle ningún aditivo químico ni natural; su riqueza en polifenoles se obtiene por ser un aceite obtenido de aceitunas recogidas de primera cosecha o cosecha temprana de una especie singularmente rica en polifenoles, en este caso la especie predominante en nuestra región, la aceituna cornicabra. Los polifenoles por tanto no son añadidos al aceite, sino que el mismo aceite los contiene de forma natural. Este aceite de recogida temprana o rico en polifenoles ha sido y es ampliamente utilizado en nuestra dieta tanto de forma doméstica como en la gastronomía por sus especiales cualidades podríamos decir que durante miles de años. La característica principal del aceite de recogida temprana aparte de su sabor

fresco es el amargor y el picor en garganta que producen curiosamente los mismos polifenoles que pretendemos estudiar.

La vía de administración será la vía bucal con el objetivo de inmunomodular la respuesta inmune local de la mucosa orofaríngea y su tejido linfoide asociado. Se administrará 2,7 mL de Aceite de oliva Cornicabra de recogida temprana rico en polifenoles, el contenido de una cucharilla de postre, vía bucal en ayunas, cada 12 horas, previo al desayuno y a media tarde. tras ingesta mantener unos segundos en cavidad oral antes de tragar. Advertirá cierto grado de amargor y picor en la garganta producido por los polifenoles que de forma natural se encuentran en el aceite de oliva.

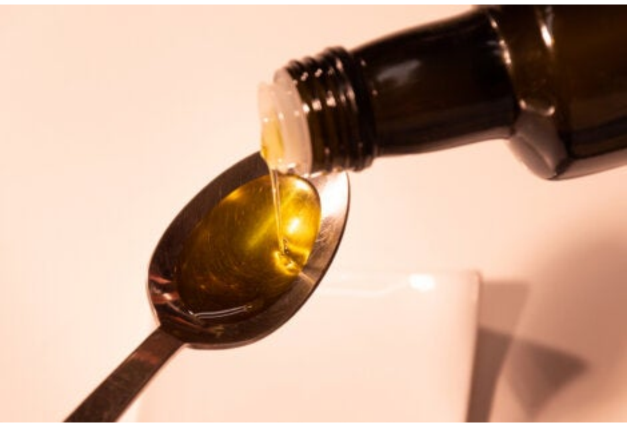

ANEXO IV: FOLLETO INFORMATIVO CON RECOMENDACIONES PARA LA PROTECCIÓN FRENTE A COVID19

Cómo protegerse y proteger a los demás

Medidas importantes para desacelerar la propagación

- Reciba la vacuna contra el COVID-19.
- Use una mascarilla para protegerse y proteger a otras personas, y detener la propagación del COVID-19.
- Mantenga una distancia de al menos 6 pies (alrededor de 2 brazos extendidos) de las personas con las que no convive.
- Evite las multitudes y los espacios con poca ventilación. Su riesgo de exposición al COVID-19 aumenta con la cantidad de gente con la que tiene contacto.

Vacúnese

- Las vacunas autorizadas contra el COVID-19 pueden ayudar a protegerlo del COVID-19.

Use mascarilla

- Todas las personas de 6 años de edad o más deben usar mascarillas en público.
- Las mascarillas se deben usar además de mantener una distancia de al menos 2 metros, especialmente si está con personas que no viven con usted.

- Si un integrante de su hogar se infecta, el resto de los miembros de su hogar deberían tomar precauciones, como el uso de mascarillas, para evitar transmitir el virus a otras personas.
- Lávese las manos o use un desinfectante de manos antes de colocarse la mascarilla.
- Use la mascarilla de tal manera que le cubra la nariz y la boca, y asegúrela por debajo del mentón.
- Ajuste la mascarilla contra los lados de su cara con las tiras detrás de las orejas, o ate los lazos detrás de su cabeza.
- Si se tiene que acomodar la mascarilla constantemente, quiere decir que no ajusta correctamente y quizá deba buscar otro tipo o marca de mascarilla.
- Debe poder respirar fácilmente.

Mantenga una distancia de 2 metros de los demás

- **Dentro de su casa:** evite el contacto cercano con las personas que están enfermas**.**
    - De ser posible, mantenga una distancia de 2 metros entre la persona enferma y otros miembros de su hogar.
- **Fuera de su casa:** mantenga una distancia de 2 metros de las personas que no viven en su hogar.
    - Recuerde que algunas personas que no tienen síntomas pueden propagar el virus.
    - Mantenga una distancia de al menos 2 metros (aproximadamente 2 brazos extendidos) de otras personas.
    - **Mantener distancia con los demás es especialmente importante para las personas que tienen mayor riesgo de enfermarse gravemente.**

Evite las grandes aglomeraciones y los espacios mal ventilados

- Estar en espacios con aglomeraciones como restaurantes, bares, gimnasios o cines aumenta su riesgo de COVID-19.
- En lo posible, evite los espacios cerrados donde no hay circulación de aire fresco del exterior.
- Si está en interiores, abra las puertas y ventanas para que ingrese aire fresco, si es posible.

Lavarse las manos frecuentemente

- Lávese las manos con frecuencia con agua y jabón por al menos 20 segundos, especialmente después de haber estado en un lugar público, o después de sonarse la nariz, toser o estornudar.

- Es de suma importancia que se lave:
  - Antes de comer o preparar la comida
  - Antes de tocarse la cara
  - Después de ir al baño
  - Después de salir de lugares públicos
  - Después de sonarse la nariz, toser o estornudar
  - Después de manipular su mascarilla
  - Después de cambiar pañales
  - Después de cuidar a una persona enferma
  - Después de tocar animales o mascotas
- Si no dispone de agua y jabón, **use un desinfectante de manos que contenga al menos un 60 % de alcohol**. Cubra toda la superficie de las manos y frótelas hasta que las sienta secas.
- **Evite tocarse los ojos, la nariz y la boca** sin antes lavarse las manos.

Cúbrase la nariz y la boca al toser y estornudar

- **Cúbrase siempre la boca y la nariz** con un pañuelo desechable al toser o estornudar o cúbrase con la parte interna del codo y no escupa.
- **Bote los pañuelos desechables usados** a la basura.
- **Lávese las manos** inmediatamente con agua y jabón por al menos 20 segundos. Si no dispone de agua y jabón, límpiese las manos con un desinfectante de manos que contenga al menos un 60 % de alcohol.

Limpie y desinfecte

- **Limpie Y desinfecte diariamente las superficies que se tocan con frecuencia.** Esto incluye las mesas, las manijas de las puertas, los interruptores de luz, los mesones, las barandas, los escritorios, los teléfonos, los teclados, los inodoros, los grifos, los lavamanos y los lavaplatos.
- **Si las superficies están sucias, límpielas.** Lávelas con agua y detergente o jabón antes de desinfectarlas.
- **Luego, use un desinfectante de uso doméstico.** Use productos seguros siguiendo las instrucciones del fabricante que figuran en la etiqueta.

Vigile su salud a diario

- **Esté atento a los síntomas.** Esté atento a la aparición de fiebre, tos, dificultad para respirar u otros síntomas **del COVID-19.**

  - Es sumamente importante si debe hacer tareas esenciales, ir a laB oficina o lugar de trabajo, y en entornos donde podría ser difícil mantener la distancia física de 2 metros.
- **Controle su temperatura** si presenta síntomas.
  - No controle su temperatura dentro de los 30 minutos posteriores a ejercitarse o después de tomar medicamentos que podrían reducir su temperatura, como el acetaminofeno.
- Siga la guía de su médico si presenta síntomas.

ANEXO V: CUADERNO DE RECOGIDA DE DATOS

Instrucciones de cumplimentación del CRD:

1º Selección del paciente a llamar: El IP suministrará un listado con todos los pacientes a contactar. Seleccionaremos el primer paciente del listado asignado. Llamaremos de manera consecutiva a todos los sujetos del listado.

2º Contactar por teléfono:

"Buenos días, mi nombre es…"

3º Preguntar: ¿Desea participar después de lo que le hemos contado? *(Si desea participar, marcaremos la casilla y continuaremos con el cuestionario)*

- ❒ Sí, deseo participar en el proyecto.

4º Preguntar: ¿Le parece bien que le realicemos una serie de preguntas para verificar su inclusión en el estudio?

¿Ha experimentado alguno de estos síntomas en los últimos días? *(En caso de marcar alguna casilla, el sujeto será excluido del estudio)*

- ❒ Fiebre/febrícula
- ❒ Tos
- ❒ Diarrea

❒ Rinorrea

❒ Odinofagia

❒ Mialgias

❒ Cansancio

❒ Cefalea

❒ Anosmia

❒ Ageusia

¿Es usted mayor de 18 años? *(Si la respuesta es NO, el sujeto será excluido del estudio)*

❒ Si

❒ No

¿Se ha realizado alguna prueba para COVID19 con resultado positivo en los últimos días? (Si la respuesta es SI, el sujeto será excluido del proyecto).

❒ Si

❒ No

5º VISITA BASAL (DÍA 2 según Plan de Trabajo): Cumplimentar los siguientes aparatados:

**ID**<br>*(Este código será suministrado por el IP)*	

- Sexo *(marcar la casilla correspondiente):*

❐ Hombre

❐ Mujer

- Fecha de nacimiento (Día/mes/año): ___ ___ / ___ ___ / 2021
- Preguntar: ¿Podría decirme cuánto mide, aproximadamente, sin zapatos? *(completar el peso en kilogramos):* ___ ___ ___ cms

❐ No sabe

❐ No contesta

- Preguntar: ¿Cuánto pesa aproximadamente, sin zapatos ni ropa? *(completar la talla en metros):* ___ ___ ___ Kg

❐ No sabe

❐ No contesta

- Preguntar: En la actualidad, ¿cuál es su ocupación-profesión?

____

______

- Grupo de riesgo *(marcar la casilla correspondiente)*:

❐ Si

❐ No

6º Indicaciones post-reclutamiento:

- Abrir el sobre correspondiente para aleatorizar a los participantes. ¿A qué grupo pertenece el paciente?

❐ Grupo control

❐ Grupo experimental

<u>7º VISITA SEGUIMIENTO:</u>

	**Día 15**	**Día 30**	**Día 60**	**Día 90**
<u>Enfermedad por coronavirus SARS-CoV-2 durante el tiempo del estudio</u>	❐ Si<br>❐ No	❐ Si<br>❐ No	❐ Si<br>❐ No	❐ Si<br>❐ No
<u>Gravedad de la enfermedad</u> *(marcar la casilla correspondient*	❐ Leve<br>❐ Moderado<br>❐ Grave	❐ Leve<br>❐ Moderado<br>❐ Grave	❐ Leve<br>❐ Moderado<br>❐ Grave	❐ Leve<br>❐ Moderado<br>❐ Grave
<u>Ingreso hospitalario por enfermedad por coronavirus SARS-CoV-2</u> *(marcar la casilla*	❐ Si<br>❐ No	❐ Si<br>❐ No	❐ Si<br>❐ No	❐ Si<br>❐ No
<u>Número de días de ingreso hospitalario</u> *(solo rellenar en caso de marcar la opción SI en el*				

Preguntar: <u>¿Ha experimentado alguno de estos síntomas en los últimos días?</u> *(marcar la casilla correspondiente)*	❒ Fiebre<br>❒ Tos<br>❒ Diarrea<br>❒ Rinorrea<br>❒ Odinofagia<br>❒ Mialgia<br>❒ Cansancio<br>❒ Cefalea<br>❒ Anosmia<br>❒ Ageusia	❒ Fiebre<br>❒ Tos<br>❒ Diarrea<br>❒ Rinorrea<br>❒ Odinofagia<br>❒ Mialgia<br>❒ Cansancio<br>❒ Cefalea<br>❒ Anosmia<br>❒ Ageusia	❒ Fiebre<br>❒ Tos<br>❒ Diarrea<br>❒ Rinorrea<br>❒ Odinofagia<br>❒ Mialgia<br>❒ Cansancio<br>❒ Cefalea<br>❒ Anosmia<br>❒ Ageusia	❒ Fiebre<br>❒ Tos<br>❒ Diarrea<br>❒ Rinorrea<br>❒ Odinofagia<br>❒ Mialgia<br>❒ Cansancio<br>❒ Cefalea<br>❒ Anosmia<br>❒ Ageusia
Eventos adversos. Preguntar: <u>¿Se ha sentido indispuesto o se ha encontrado mal después de empezar a</u>	❒ Si<br>❒ No	❒ Si<br>❒ No	❒ Si<br>❒ No	❒ Si<br>❒ No
<u>Describir el evento adverso</u> (solo rellenar en caso de marcar la opción SI en el				

<u>Causa de la retirada del sujeto</u> (solo rellenar en caso de que sea necesario)	❒ Toxicidad relacionada con el fármaco.<br>❒ Embarazo.<br>❒ Incumplimiento de los plazos establecidos.<br>❒ Pérdida de seguimiento.	❒ Toxicidad relacionada con el fármaco.<br>❒ Embarazo.<br>❒ Incumplimiento de los plazos establecidos.<br>❒ Pérdida de seguimiento.	❒ Toxicidad relacionada con el fármaco.<br>❒ Embarazo.<br>❒ Incumplimiento de los plazos establecidos.<br>❒ Pérdida de seguimiento.	❒ Toxicidad relacionada con el fármaco.<br>❒ Embarazo.<br>❒ Incumplimiento de los plazos establecidos.<br>❒ Pérdida de seguimiento.
<u>Vacunado para COVID19</u> (marcar la casilla	❒ Si<br>❒ No	❒ Si<br>❒ No	❒ Si<br>❒ No	❒ Si<br>❒ No
<u>Número de dosis</u> (solo rellenar en caso de marcar la opción SI en				

8º <u>PREGUNTAR SOLO EL DÍA 90 DE SEGUIMIENTO AL GRUPO EXPERIMENTAL:</u> ¿Ha completado el tratamiento propuesto? *(marcar la casilla correspondiente)*

❒ Si

❒ No
